# The genetic landscape and clinical implication of pediatric Moyamoya angiopathy in an international cohort

**DOI:** 10.1038/s41431-023-01320-0

**Published:** 2023-04-04

**Authors:** Paolo Zanoni, Katharina Steindl, Heinrich Sticht, Beatrice Oneda, Pascal Joset, Ivan Ivanovski, Anselm H. C. Horn, Elena M. Cabello, Julia Laube, Markus Zweier, Alessandra Baumer, Anita Rauch, Nadia Khan

**Affiliations:** 1 Institute of Medical Genetics, University of Zürich, Schlieren-Zurich, 8952 Switzerland; 2 Institute of Biochemistry, Friedrich-Alexander-Universität Erlangen-Nürnberg (FAU), Erlangen, 91054 Germany; 3 Zurich Center for Integrative Human Physiology, University of Zurich, Zurich, 8000 Switzerland; 4 Neuroscience Center Zurich, University of Zurich and ETH Zurich, Zurich, 8000 Switzerland; 5 Moyamoya Center, University Children’s Hospital, University of Zurich, Zurich, 8032 Switzerland

**Keywords:** Stroke, Disease genetics

## Abstract

Pediatric Moyamoya Angiopathy (MMA) is a progressive intracranial occlusive arteriopathy that represents a leading cause of transient ischemic attacks and strokes in childhood. Despite this, up to now no large, exclusively pediatric MMA cohort has been subjected to systematic genetic investigation. In this study, we performed molecular karyotyping, exome sequencing and automated structural assessment of missense variants on a series of 88 pediatric MMA patients and correlated genetic, angiographic and clinical (stroke burden) findings. The two largest subgroups in our cohort consisted of *RNF213* and neurofibromatosis type 1 (NF1) patients. While deleterious *RNF213* variants were associated with a severe MMA clinical course with early symptom onset, frequent posterior cerebral artery involvement and higher stroke rates in multiple territories, NF1 patients had a similar infarct burden compared to non-NF1 individuals and were often diagnosed incidentally during routine MRIs. Additionally, we found that MMA-associated *RNF213* variants have lower predicted functional impact compared to those associated with aortic disease. We also raise the question of MMA as a feature of recurrent as well as rare chromosomal imbalances and further support the possible association of MMA with *STAT3* deficiency. In conclusion, we provide a comprehensive characterization at the genetic and clinical level of a large exclusively pediatric MMA population. Due to the clinical differences found across genetic subgroups, we propose genetic testing for risk stratification as part of the routine assessment of pediatric MMA patients.

## Introduction

Moyamoya angiopathy (MMA) is a progressive intracranial occlusive arteriopathy characterized by a stenosis of the distal portion of the internal carotid artery and involvement of the anterior, middle, and posterior cerebral arteries. To compensate for the reduction in blood flow, the brain develops a compensatory angiogenetic response, thus producing an intracerebral network of small-caliber collaterals, which is responsible for a cloud-like appearance (“Moyamoya” in Japanese) in angiograms [1]. Due to progressive reduction in brain perfusion, pediatric patients with MMA can suffer transient ischemic attacks (TIA) and strokes, representing the most common clinical presentations [2]. Less common manifestations include seizures, headaches, choreiform movements, cognitive or psychiatric problems and, rarely, brain hemorrages [1]. MMA has been frequently described in Japan, where it represents the most common pediatric cerebrovascular disease (prevalence and incidence up to 10.5:100,000 and 0.94:100,000, respectively) [3], and, with up to 18 times lower incidence [4], also in individuals of American and European descent, although this could be due to lower disease awareness among clinicians. Interestingly, female individuals are affected more frequently compared to males [3].

Although MMA represents an isolated manifestation in otherwise healthy individuals in approximately 50-75% of cases, it has also been found enriched in conjunction with a number of diverse conditions such as neurofibromatosis type 1 (NF1), sickle cell disease, Trisomy 21, congenital cardiac anomalies, renal artery stenosis and giant cervicofacial hemangiomas [1, 5]. Such MMA cases associated with other conditions are labeled as Moyamoya syndrome (MMS), while non-syndromic MMA is called Moyamoya disease (MMD).

A genetic component for MMD has been suggested for a long time based on the different frequencies between ethnicities, the presence of numerous familial cases and the higher risk in twins [6]. Besides earlier linkage analyses based on microsatellite markers, two GWAS studies to identify Moyamoya susceptibility loci have been conducted [7]. These, combined with broad sequencing efforts [6] and traditional family studies, revealed, among others, the role of *RNF213*, a gene encoding for the largest E3-uibiquitin transferase in the human proteome [8]. Despite the increasing amount of studies on the function of *RNF213*, its mechanistic link with MMA remains elusive, as its involvement in multiple different processes such as proliferation and inflammatory signaling in endothelial cells, vascular stability and pruning, apoptosis, response to hypoxia, and, more recently, lipid and glucose metabolism and response to lipotoxicity has been suggested to play a pathogenetic role [9].

The recurrent *RNF213* variant p.(Arg4810Lys) (rs112735431) represents the most common genetic abnormality found in Japanese, Korean and Chinese MMA patients [10] (with minor allele frequency [MAF] of 1.15% in Koreans within the GnomAD database) and has been associated with an increased risk to develop MMA (the average OR was 96.5 (95%CI: [67–139]) [11] and 49.4 (95%CI: [37.2-65.6]) [12] in two large meta-analyses). The p.(Arg4810Lys) variant is not present in the European population (*N* = 129162 non-Finnish Europeans in the GnomAD database), although other *RNF213* variants clustering in the gene’s RING domain may predispose to MMA in Europeans [13].

Despite the evolving evidence for the role of *RNF213* in MMA patients of European ancestry, most of the studies focused on adult populations, with a few mixed adult/pediatric studies confirming the role of *RNF213* variants in children [14, 15] as well as a few additional studies reporting rare syndromic causes of MMA [16–20]. As for many other disorders, pediatric MMA cases are likely enriched for strong causative genetic factors, since environmental factors have had less time to play a significant role. Since no large, exclusively pediatric MMA cohort has been subjected to systematic genetic investigation, we performed a comprehensive genotype-phenotype study using whole-exome sequencing (WES) as well as chromosomal microarray analysis (CMA) in a clinically well-characterized large pediatric MMA cohort (*N* = 88).

## Subjects and methods

### Patients

**Fig. 1:**
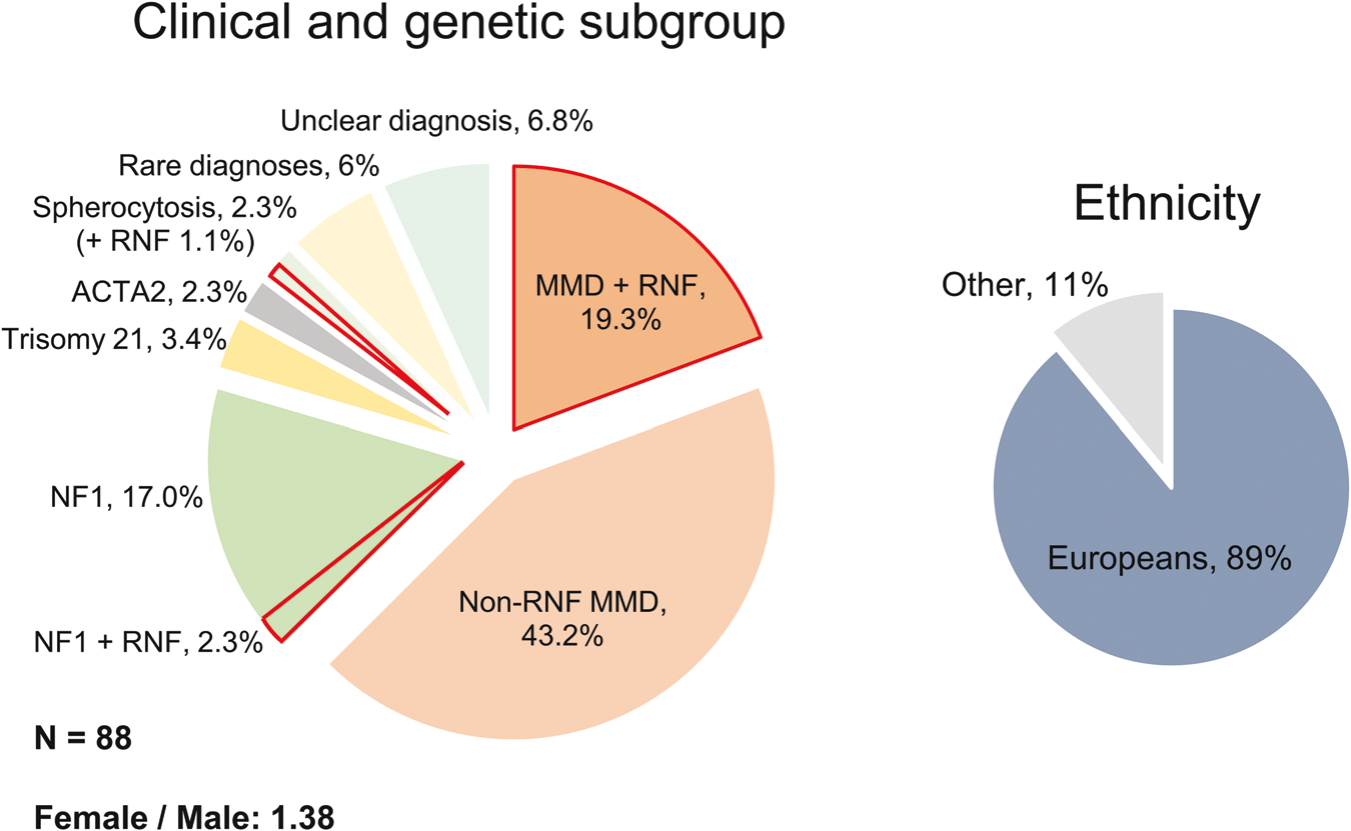
Clinical presentation and genetic subgroups of the study cohort. Main clinical and genetic subgroups (left) and ethnicity (right). The percentage of cases with predicted deleterious and possibly deleterious *RNF213* variants (as defined in Fig. 2B) are highlighted in red and labeled as “*RNF”*. In the left panel, “rare diagnoses” were SHAM syndrome, Alagille syndrome, MOPD type 2 syndrome, *STAT3* deficiency and brain small vessel disease type 2. “Unclear diagnosis” refers to MMS patients for whom neither a clinical nor a genetic diagnosis associated with MMA could be established.

88 pediatric MMA patients treated at the Moyamoya Center of the Children Hospital Zurich, Switzerland, from 2011 to 2020 were included in the analysis. Eighty-nine percent (*N* = 78) of the affected individuals were of European descent (Fig. 1). After initial screening with magnetic resonance imaging / angiography (MRI/MRA), the diagnosis was confirmed by six-vessel cerebral angiograms. All patients showing hemodynamic insufficiency based on H2^15^O-PET at baseline and after acetazolamide challenge underwent one or more staged cerebral revascularization surgeries.

MMD was defined as isolated MMA but also contains 13 patients either with other associated anomalies that, however, are not representing a defined syndrome or exhibiting blended phenotypes with independent disorders such as extracranial vascular anomalies or thrombophilia due to Factor V-Leiden. Patients that presented with MMA in combination with a known or suspected syndromic disorder were categorized as having Moyamoya Syndrome (MMS). Similar criteria have been recently employed by others [13] and are based on the notion that, while additional extracranial vascular manifestations may increasingly be recognized to be part of the MMD spectrum [21], other isolated congenital anomalies as well as common variants such as Factor V-Leiden may just represent chance associations and do not suffice to classify a case as syndromic.

The age at onset was defined as the age at first clinical manifestation clearly attributable to MMA.

### Analysis of the preoperative angiographic and cerebral MRI data

Stroke burden was evaluated on the initial T2- and FLAIR-MRI images and divided into territorial i.e ACA (anterior cerebral artery), MCA (middle cerebral artery) or PCA (posterior cerebral artery) and watershed (ACA-MCA and MCA-PCA) distribution and, if present, in both the cortical and subcortical areas. Six-vessels cerebral angiograms were analyzed for involvement (stenosis/occlusion) of the respective cerebral arteries (ACA, MCA, PCA).

### Genetic analysis

WES was performed on blood-derived DNA as previously described [22]. Briefly, 59 samples (“phase 1”) were sequenced on an Illumina HiSeq 2500 (Illumina, Zurich, Switzerland) after exon capture with the IDT xGen Exome Research Panel v1 (Integrated DNA Technologies, Coralville, Iowa, USA). The remaining 29 samples (“phase 2”) were sequenced on an Illumina NovaSeq 6000 after exon capture with the IDT xGen Exome Research Panel v2. High-quality variants with a global MAF lower than 2% were annotated from VCF files using the wAnnovar online tool (http://wannovar.wglab.org/). Further variant filtering was performed using R (https://www.r-project.org/). A (candidate) gene filter (Table S1) was then applied that contained all 87 genes associated until now with MMA based on a literature research as well as hits from the largest GWAS study [23]. For individuals with a known or suspected genetic disorder not included in the MMA gene filter, the respective known disease genes were tested additionally. For those cases where at least one parent was available, parental inheritance of possibly clinically relevant CNVs as well as all *RNF213* and *NF1* variants was analyzed by chromosomal microarray and WES (see Tables S2, S4 and S5). *RNF213* and *NF1* variant coordinates refer to transcripts NM_001256071.3 and NM_000267.3, respectively.

Classification of potentially deleterious variants followed the ACMG guidelines [24] and was performed in an automated fashion using the Intervar tool (https://wintervar.wglab.org/).

For *RNF213*, CADD scores [25] were combined with VIPUR scores [26] to stratify variants by predicted deleteriousness. While CADD scores provide an integrated score based on 60 different annotations [25], VIPUR scores include an automated assessment of the structural effects [26]. CADD and VIPUR scores were first clustered based on the expectation-maximization (EM) method as implemented by the R *mclust* library (https://CRAN.R-project.org/package=mclust). Specifically, univariate clustering was applied to each score using an unequal variance model with two expected mixture components. We then classified the variants in the “high score” clusters for both CADD (24 or higher) and VIPUR (0.46 or higher) as “deleterious”, those in the “high score” cluster only for either CADD or VIPUR as “possibly deleterious” and the rest as “benign”. *RNF213* variants previously published in the context of vascular disease were obtained from the Human Gene Mutation Database, http://www.hgmd.cf.ac.uk/ac/index.php, accessed: August 11th 2020. Online databases such as ClinVar (https://www.ncbi.nlm.nih.gov/clinvar/) and LOVD (https://www.lovd.nl/) were used to evaluate *NF1* variants.

A search for deep intronic *NF1* variants in individuals 94488 and 95540 was performed by whole-genome sequencing using the TruSeq PCR-free workflow on a NovaSeq 6000 (Illumina incorp.). CMA was performed on blood-derived DNA as previously described [22].

### Statistics

**Fig. 2:**
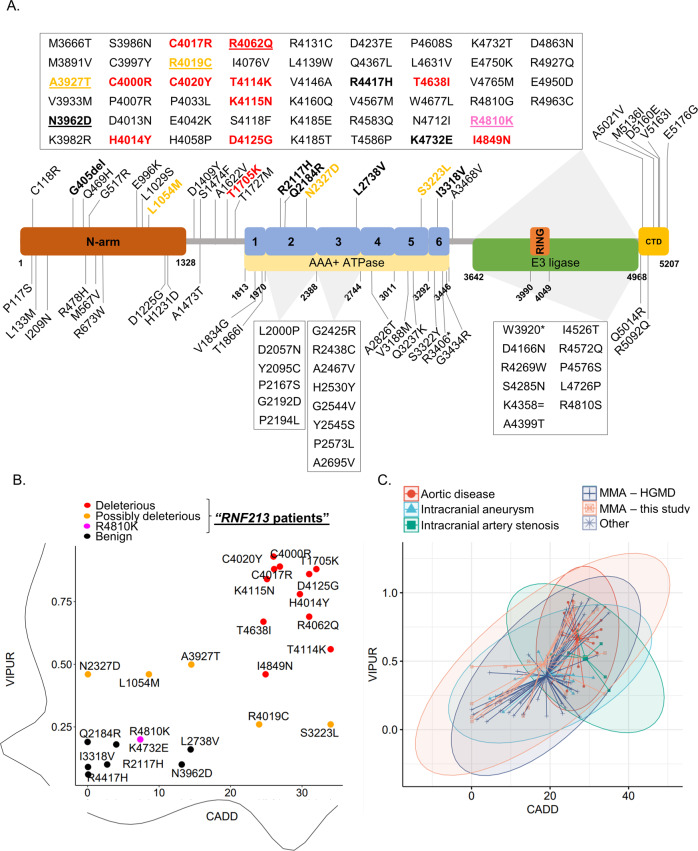
Location and pathogenicity prediction of *RNF213* variants. **A** Variant location. The diagram shows the structure of the *RNF213* gene together with the 25 variants identified in our study population (in bold; colored for pathogenicity scores as in panel 2B) as well as previously reported variants detected either in patients with MMA /intracranial arterial stenoses (above) or with other arteriopathies (below; source: HGMD (http://www.hgmd.cf.ac.uk/ac/index.php)). Previously reported variants that have been detected in our study population as well are underlined. Amino acid coordinates refer to transcript NM_001256071.3; for simplicity, the one-letter code was used to symbolize amino acids; for HGVS-conform variant nomenclature, refer to Tables S3 and S4; domain locations were derived from Ahel et al. [8]. N-arm: N-terminal arm; RING: RING finger domain; CTD: C-terminal domain. **B** Variant classification based on the combination of CADD and VIPUR scores. All variants detected in our cohort except G405del, which could not be tested using the VIPUR algorithm, are shown. Density distribution of CADD and VIPUR scores are displayed on the side of each respective axis. Data points were classified by expectation-maximization as described in the methods. Please refer to the legend on top of the graph for color coding of the variants. All heterozygotes for predicted deleterious and possibly deleterious variant are described as “*RNF213* patients” in this paper. **C** Comparison with previously reported *RNF213* variants. The VIPUR and CADD scores of 90 previously reported *RNF213* variants (source: HGMD) are shown here together with the variants detected in this study (see **A**; labeled as “MMA – this study”). Data points were colored based on the reported clinical phenotype. For 11 previously reported variants no VIPUR score could be generated either because they were located in the N-terminal region (no structural information available; 8 variants) or they were nonsense (2 variants) or synonymous (1 variant). Each point represents one variant. Segments represent the distance between each variant and the respective group’s average. Ellipses represent data distribution in each group assuming a multivariate normal distribution with a confidence threshold of 95%.

**Fig. 3:**
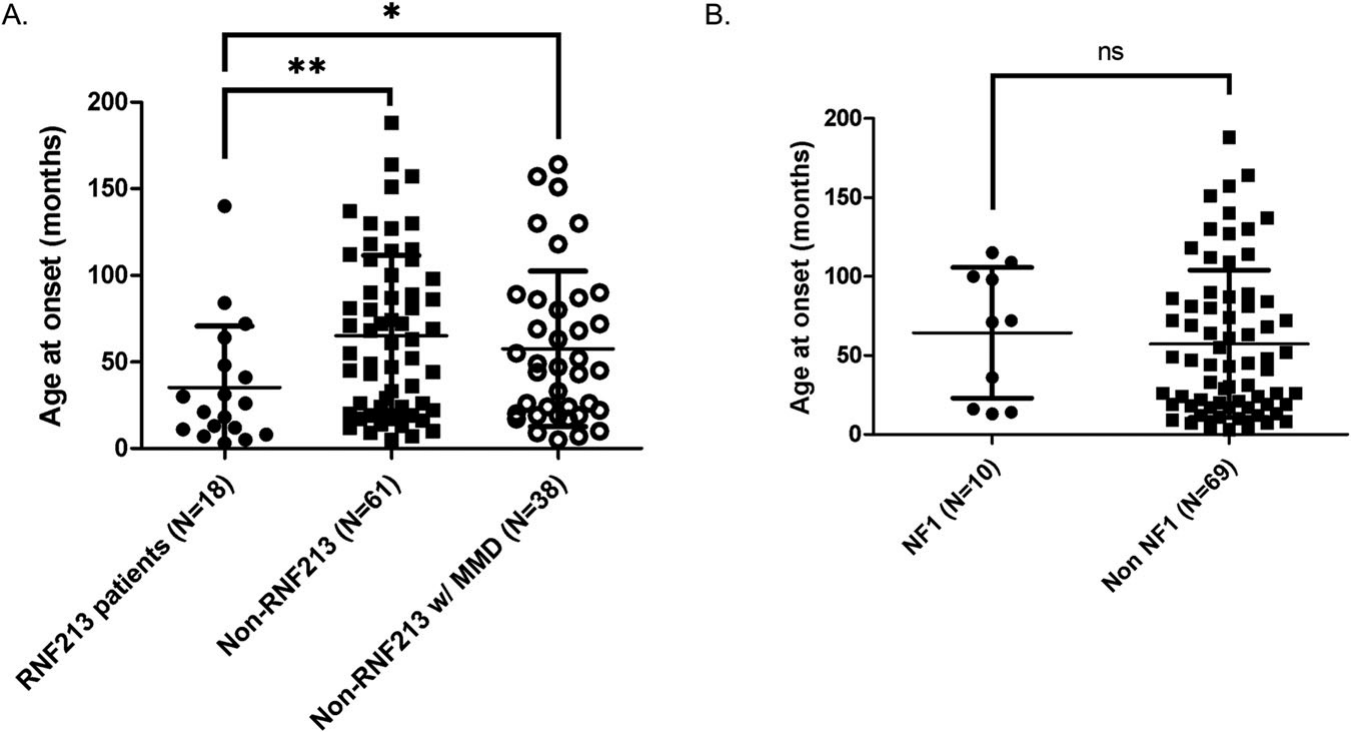
Correlation between genotype and age at onset in our cohort. **A** Age at MMA symptom onset for *RNF213* patients compared to all non-*RNF213* as well as to all non-*RNF213* with MMD. **B** Age at MMA symptom onset for patients with a clinical diagnosis of NF1 compared to all other patients in our cohort. Patients diagnosed by MRI in the asymptomatic phase as well as NF1 patients with additional *RNF213* (possibly) deleterious variants were excluded from this analysis. Lines and whiskers represent mean ± SD. ns non-significant, *p < 0.05, **p < 0.01 by two-tailed Mann–Whitney test.

**Fig. 4:**
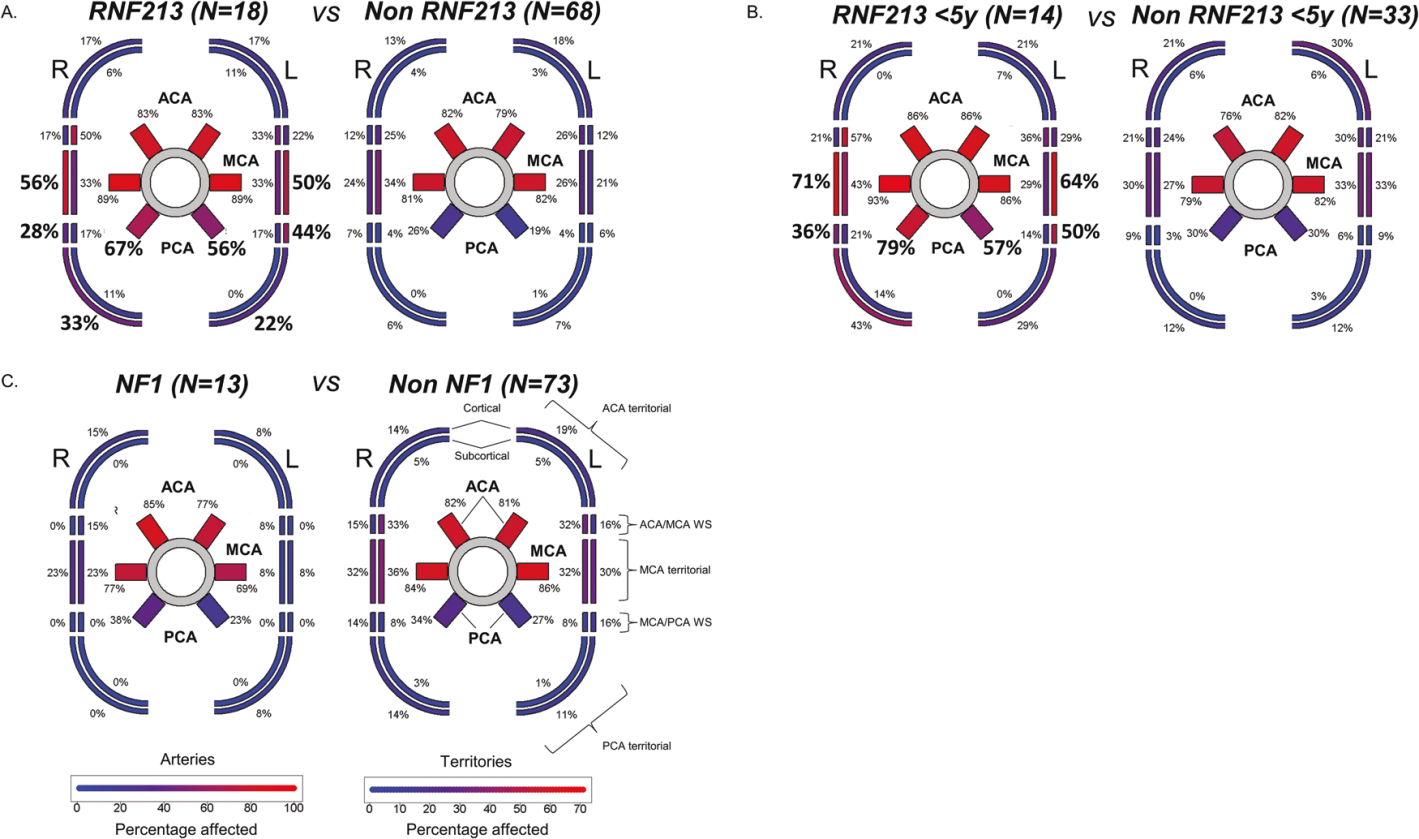
Genotype–phenotype correlation for angiographic and MRI findings. Schematic representation of the Circle of Willis and the respective brain territories supplied by the anterior, middle and posterior cerebral artery (ACA, MCA and PCA). Arteries involved and their respective territories are colored based on percentage of patients affected, as reported in the legend at the bottom. The involvement of each artery and territory was compared between *RNF213* patients (**A**), *RNF213* patients below the age of 5 (**B**), NF1 patients (**C**) and the respective rest of the cohort by Fischer’s Exact Test (FET) irrespective of laterality. Significant differences (FET *p* value <0.05 after Bonferroni correction) are shown in bold. Patients aged below 5 years (**C**) were separately analyzed in order to exclude the possible confounding effect of age, as *RNF213* patients are younger than non-*RNF213* ones (Fig. 3A). Two NF1 patients (68632 and 82059) were excluded from this analysis as they carried an additional (possibly) deleterious *RNF213* variant as well.

Descriptive statistics for our patient cohort were reported as percentages for categorical variables and means plus standard deviations for continuous variables. The different subgroups in Figs. 2C and 3 were compared by computation of the exact *p* values using a two-tailed Mann-Whitney test. The graphs in Fig. 3 were generated using GraphPad Prism version 9 for Windows (GraphPad Software, LaJolla, CA, USA). In Fig. 4, frequencies across subgroups were compared by Fisher’s exact test (FET) with Bonferroni correction without taking laterality into account. The laterality of vascular involvement across groups was compared by FET. A significance threshold of 0.05 was chosen for all tests. All plots and analyses in Figs. 2B, C and 4 were generated and performed with R (https://www.R-project.org/) with the addition of the *grid*, *gridExtra* and *ggpubr* packages. All other statistical analyses were performed with GraphPad Prism (see above).

## Results

### Genetic analysis of a pediatric Moyamoya population

Demographic and clinical characteristics of the 88 pediatric MMA patients are shown in Fig. 1.

Eighty patients (91%) presented with ischemic symptoms (mean age at diagnosis 57.2 ± 46.2 months), while 8 (9%) were diagnosed with MMA incidentally when brain MRI was performed because of a different underlying disorder (mean age 90.6 ± 64.9 months). Thirty-three patients had MMS (37.5%), while the remainder had MMD (*N* = 55, 62.5%). None of the parents of the patients were affected by MMA.

Detailed CMA results and their interpretation are available as Table S2. Besides three patients with Trisomy 21, four other children were found to carry (possibly) pathogenic copy number variants (CNVs). These included a rearrangement in Xq24 associated with SHAM (severe hemophilia A and Moyamoya) syndrome [17] (ID76728), the recurrent 16p11.2 deletion [27] together with a 2.1 Mb copy number gain (4 copies) in 1q21.1-21.2 [28] (ID73732) as well as the recurrent 15q11.2 BP1-BP2 duplication [29] (ID69946). Patient 76654, a girl with hypoplastic bulbus oculi whose mother had arrhythmias and whose sister died at age 9 because of a pontine glioma carried the maternally inherited deletion in 1p36.22, encompassing among others the CASZ1 and MTOR genes. Except for SHAM syndrome, none of the CNVs mentioned above have been reported in MMA patients to date.

Sequence variants found by WES in MMA-associated genes are listed in detail in Table S3. A total of 25 *RNF213* (NM_001256071.3) variants were detected in 27 individuals (Fig. 2A and Table S4). Of these, 6 individuals carried more than one variant. Predictions of deleteriousness by CADD and VIPUR scores are summarized in Fig. 2B and Table S4. The p.(Gly405del) variant could not be tested using the VIPUR algorithm and was thus classified as non-deleterious based on its CADD score (12.8).

One patient whose mother was of Korean descent carried the recurrent Asian p.(Arg4810Lys) variant [10]. Notably, this p.(Arg4810Lys) variant would not have been classified as deleterious by CADD and VIPUR scores, as expected for algorithms aimed at identifying highly penetrant variants. Nevertheless, in vitro and in vivo data, albeit at times conflicting, support its pathogenicity [6].

With the exception of the p.(Thr1705Lys) and p.(Thr4638Ile) variants (GnomAD global MAF: 0.0043 and 0.0007, respectively), no other *RNF213* variant predicted deleterious by our algorithm was present in the GnomAD database. Of the aforementioned (possibly) deleterious *RNF213* variants, only p.(Arg4810Lys), p.(Arg4062Gln), p.(Arg4019Cys) and p.(Ala3927Thr) have been previously reported in individuals with MMA (source: HGMD; Table S4). One patient carried the previously reported p.(Asn3962Asp) [30] variant, which was predicted to be benign by our threshold. As this report did not provide direct proof of deleteriousness of this variant, we did not reclassify it as deleterious in this study. Finally, the p.(Asn2327Asp) variant has been identified in several multiple sclerosis families [31], although the validity of this publication has been disputed [32]. Among (possibly) deleterious *RNF213* variants, parental inheritance could be established in 16/23 occurrences and was *de novo* in only 2/16 cases (13%).

As shown in Fig. 2C, we found that previously reported *RNF213* variants reported in patients with aortic pathology were associated with the highest average CADD and VIPUR scores. This difference was statistically significant when compared to variants in MMA patients (*p* = 0.003 and 0.0007 for CADD and VIPUR scores, respectively).

In line with previous reports in (mostly adult) Europeans [13, 14], 10 out of the 11 *RNF213* variants predicted deleterious by our algorithm lie in the E3-ligase domain (Fig. 2A).

Among the 20 patients with predicted deleterious and possibly deleterious *RNF213* variants (referred to as “RNF213 patients”, Table S4, Fig. 2B), 17 presented with MMD (31% of all MMD patients; Fig. 1, left). The three remaining patients had MMS, two with NF1 and one with spherocytosis. The difference in the proportion of patients with *RNF213* (possibly) deleterious variants with MMD vs MMS was statistically significant (*p* = 0.0196, OR = 4.4, 95% CI [1.1–25.6] by FET). Among *RNF213* patients with MMD, patient 73577, a girl carrying the maternally inherited p.(Thr1705Lys) and p.(Leu1054Met) variants in *cis*, presented with mid-aortic syndrome, a rare form of aortic coarctation which has been recently linked to *RNF213* as well as *NF1* variants [33]. No other pathogenic variant in genes related to arteriopathies was detected in this patient. The only other patient with aortic pathology was patient 90549, with aortic isthmus stenosis, in whom we did not find a causative variant for MMA. However, further exome analysis revealed the maternally inherited p.(Gly624Asp) known pathogenic variant in *COL4A5*, causing X-linked Alport syndrome, which was also linked to aortic abnormalities in male patients [34]. This patient also carried the maternally inherited p.(Leu2707ProfsTer8) variant in CHD6, a candidate gene for developmental disorders. Nevertheless, this variant lies at the 3′ end of the gene’s coding sequence, is predicted to escape nonsense-mediated decay and neither the mother nor the child had intellectual disability.

As reported in Fig. S1 and Table S5, in 11 out of 15 patients suspected of having NF1 a pathogenic or likely pathogenic *NF1* (NM_000267.3) variant was identified by WES, while in 2 additional patients, the presence of an intragenic deletion was revealed by MLPA. Whole-genome sequencing allowed additionally the detection of the pathogenic c.6579 + 18A > G variant in individual 95540, while no clear pathogenic variant was found in patient 94488. Further analysis of the exome data for this patient did not reveal any variants in *SPRED1*, *NF2* or other genes associated with RASopathies that could explain the phenotype. Conventional karyotyping was negative as well. Only two additional *NF1* variants, namely p.(Thr2486Ile) and p.(Ala2511Val), both known to be benign or likely benign based on online databases such as Clinvar and LOVD were found in clinically unaffected individuals.

Among other MMA-associated genes, pathogenic variants were found in *ACTA2* (2 patients, both carrying the p.(Arg179His) variant), *GUCY1A3* (Moyamoya 6 with achalasia, OMIM 615750, AR), *JAG1* (Alagille syndrome, OMIM 118450, AD)*, ANK1* and *SPTB* (spherocytosis) and *PCNT* (microcephalic osteodysplastic primordial dwarfism, type II / MOPD type II, AR, OMIM 210720). Both ACTA2 patients in our cohort presented the typical angiographic pattern for this syndrome, which has been classified by some authors as representing a distinct form of cerebral arteriopathy from MMA [35].

Additionally, a female patient with autoimmune hemolytic anemia, pure red cell aplasia, thrombocytopenia, short stature and MMA was found to carry the *de novo* recurrent p.(Pro714Leu) variant in *STAT3*. This variant affects the last nucleotide of exon 22 of *STAT3*. In silico tools predicted a 9.2% decrease in the efficiency of the adjacent donor site.

### Genotype–phenotype analysis

Possible genotype–phenotype correlations were analyzed concerning the age at onset of symptoms, the number of arteries involved, and the number of ischemic areas/strokes present amongst the different subgroups.

Nine patients were excluded from the analysis of age at onset. Of these, 8 patients (4 of whom had NF1; 1 with NF1 and an additional *RNF213* predicted deleterious variant [82059]) received an incidental MMA diagnosis at MRI while one (68632) was a symptomatic NF1 patient who carried an additional possibly deleterious *RNF213* variant as well.

MMD patients were on average 24.9 months (2.1 years) younger at onset compared to MMS patients (50.7 ± 43.4 versus 75.7 ± 47.4 months, respectively; *p* = 0.0277).

As visible in Fig. 3A, *RNF213* patients were on average 1.8 times as young at onset compared to non-*RNF213* individuals (35.2 ± 35.5 versus 65.1 ± 46.5 months, *p* = 0.0078) and 1.6 times younger than non-*RNF213* patients with MMD (*p* = 0.0418). The proportion of individuals with (possibly) deleterious *RNF213* variants as well as the age at onset did not differ significantly by sex or when the area of origin (norther, central, southern Europe as well as non-Europe) was considered. Of notice, no deleterious *RNF213* variants were detected in the 8 non-European patients.

No significant difference in the age at onset between *NF1* patients and all non-NF1 individuals in the cohort was present (Fig. 3B, *p* = 0.5603). Although *RNF213* patients were on average younger at onset than NF1 patients (35.2 ± 35.5 versus 64.4 ± 41.3 months), this difference did not reach statistical significance as a result of incidentally diagnosed patients being excluded from the analysis (*p* = 0.0521).

In the overall cohort (*N* = 88), ACA and MCA were the most commonly involved vessels (81.5 and 83%, respectively), while ischemic areas localized most frequently in the cortical (29%) and subcortical (31%) MCA territories. On the contrary, the PCA was involved on average in 32% of cases. Both NF1 patients with (possibly) deleterious *RNF213* variants, in whom PCA involvement was also seen, were excluded from further analysis of arterial involvement and strokes.

While involvement of the anterior circulation (Fig. 4A) was not significantly different across groups, PCA involvement was significantly higher (61.5% vs 22.5% on average, OR = 5.3, 95% CI [2.3–12.6], *p* = 3.5 × 10^−4^, Fig. 4A) and more often bilateral (50% vs 16%, *p* = 4.9 × 10^-3^) in *RNF213* patients compared to the non-*RNF213* group. This difference in PCA involvement resulted in a higher number of total ischemic areas (5.2 ± 4.5 vs 2.5 ± 3.0, *p* = 4 × 10^-3^), with cortical PCA territorial, cortical MCA-PCA watershed and cortical territorial MCA strokes (OR = 5.4, 7.8, 3.9; 95%CI: [1.8–16.6], [2.7–23.5], [1.7–9.1], *p* = 1.5 × 10^−2^, 3.4 × 10^−4^ and 8.7 × 10^−3^ respectively, Fig. 4A) being significantly more affected in *RNF213* patients.

As *RNF213* patients are younger than non-*RNF213* ones, the possible confounding effect of age was excluded by comparing the angiographic and stroke data only in *RNF213* and non-*RNF213* patients below 5 years (60 months) of age. PCA arterial involvement as well as MCA cortical territorial and MCA-PCA cortical watershed strokes remained significant in this age subgroup as well (OR = 4.8, 4.4 and 7.3, 95%CI [1.7–14.2], [1.6–13.2], [2.1–27.7], *p* = 1.5 × 10^−2^, 2.0 × 10^−2^ and 4.5 × 10^−3^, respectively, Fig. 4B).

In contrast to *RNF213* patients, NF1 patients did not show any significant difference in their angiographic and stroke patterns as well as in the frequency of bilateral involvement compared to non-NF1 patients (Fig. 4C). Nevertheless, they presented with the lowest average number of ischemic areas per patient compared to all other subgroups (1.2 ± 1.5). This value was less than half of that of *RNF213* patients (5.2 ± 4.5, *p* = 0.0009) and tended to be lower when compared to all other MMS patients (3.0 ± 2.4, *p* = 0.0222).

Similar to the findings for age at onset, restricting the angiographical and stroke pattern analysis of *RNF213* to MMD patients only, the differences shown in Fig. 4A were reproducible, albeit with lower significance (data not shown).

## Discussion

Our comprehensive analysis in an unselected exclusively pediatric sample shows that *RNF213* patients and neurofibromatosis patients represented the two largest subgroups. A comparison of the relative frequencies with previous large sequencing studies is hampered by differences in the probands’ age as well as in the inclusion criteria used. In particular, no shared consensus exists on the definition of MMD and MMS, with the presence of common genetic or clinical findings such as Factor V-Leiden having been considered sufficient by other authors to classify a patient as having MMS. While the prevalence of *RNF213* deleterious variants in our MMD patients (31%) lies either above or in line with what was detected in mixed pediatric/adult populations [14, 15], Guey et al. [13], found *RNF213* rare coding variants in 48% of their smaller (*N* = 21) sub-cohort of pediatric and familial cases. Another significant difference with previous studies resides in the in silico methods used to evaluate the functional effect of *RNF213* variants, as we refined our predictions by incorporating structural evaluations in the form of VIPUR scores. Interestingly, only 4 out of 17 (possibly) deleterious *RNF213* variants detected in our cohort had been previously described in patients with MMA or intracranial vascular stenoses, thus highlighting the very high variant heterogeneity in non-Asians. The low frequency of *de novo* occurrence of *RNF213* (possibly) deleterious variants in our cohort points at incomplete penetrance. Previous segregation studies estimated the penetrance of variants other than p.(Arg4810Lys) to be about 25% [13], although these may represent overestimations due to ascertainment bias. As suggested by the work of Ihara [36] and Ikeuchi [37], it is possible that variants in downstream targets of *RNF213* such as FLNA and NFAT1 may modulate the penetrance of the MMA phenotype.

Pediatric patients carrying deleterious *RNF213* variants represent a clinically and angiographically distinct subgroup within our cohort, with much (1.8 times) earlier age at symptom onset, frequent bilateral PCA involvement and higher frequency of strokes in the MCA and PCA territories. Notably, a large study (*N* = 1385) [38] investigating artery involvement in mostly adults carrying the East-Asian *RNF213* p.(Arg4810Lys) variant found that heterozygotes are significantly younger and with more frequent PCA involvement compared to patients without *RNF213* variants, although no significant differences were noted in stroke and TIA frequencies. Since the clinical expression of this variant is much lower than for the variants found in our pediatric cohort, the p.(Arg4810Lys) variant might be considered a hypomorphic allele. In fact, despite the in vitro and in vivo data supporting the pathogenicity of p.(Arg4810Lys) [6]¸ the at times contradictory functional findings as well as the very high frequency of this variant in East-Asians suggest a mild effect at most.

In the last few years, the p.(Arg4810Lys) variant has been associated with other intra- and extracranial vascular manifestations as well, thus suggesting that it could lead to a wider phenotypic spectrum than previously recognized [21]. Among others, homozygosity for p.(Arg4810Lys) has been reported in association with pulmonary artery stenosis, pulmonary hypertension as well as extracranial artery stenosis [21]. Nevertheless, in our cohort only one patient carrying two maternally inherited *RNF213* variants in cis had mid-aortic syndrome (73577), indicating that extracranial arterial involvement may either represent chance associations or a mutation-specific phenotype. In support of the latter hypothesis, our in silico analysis of previously published *RNF213* variants suggests that aortic vascular manifestations may only present in heterozygotes for more deleterious variants.

Although MMA became clinically manifest in NF1 patients on average later than in *RNF213* patients, no differences in age at onset of MMA and infarct burden were seen compared to non-NF1 patients; thus we could not directly confirm previous suggestions that NF1-associated MMA follows a milder course with lower infarct burden [39]. Nevertheless, this difference may be explained by the fact that 26% of the NF1 cases were detected by imaging in the asymptomatic phase. Additionally, as suggested by the PCA involvement in two individuals in our series and reported in the past [40], the presence of variants in modifier genes such as *RNF213* could lead to a more severe phenotype. Because of this MRAs should be taken into consideration as part of the standard of care in pediatric NF1 patients.

Furthermore, for several recurrent and rare CNVs we raise the question of association with MMA. Ouellette and colleagues showed recently how haploinsufficiency of the murine 16p11.2 ortholog leads to altered angiogenesis as well as functional cerebrovascular anomalies in vivo and human-derived endothelial cells display faulty angiogenetic activity [41]. *CASZ1*, which is located in 1p36.22, plays a role in the immune system [42], acts as a tumor suppressor gene involved in brain cancer [43], and truncating variants in this gene have been associated with dilatative and left ventricular non-compaction cardiomyopathy [44]. Additionally, *CASZ1* is expressed in the vasculature and was identified as a risk locus for blood pressure and stroke [45], so that it seems reasonable to speculate that the cardiac phenotype in the mother, the pontine glioma in the sister as well as MMA in the index case may represent different manifestations of the same genetic defect. Although more studies are needed to underpin the causality of these associations, our findings point towards an added value of copy number analysis in pediatric MMA patients.

Moreover, we provide an additional report of *STAT3* deficiency associated with MMA [46], thus strengthening the evidence in support of this gene’s possible role in the pathogenesis of this condition. Monies and colleagues provide the first report of this association [46] and argue that truncating mutations may be responsible for it. Nevertheless, Mauracher and colleagues [47], who first described the immuno-hematological phenotype of our patient, functionally characterized the p.(Pro714Leu) variant (also known as p.(Pro715Leu)) as leading to STAT3 gain-of-function, albeit without taking its possible effect on splicing into consideration. Despite in silico tools showing only a modest effect on splicing efficiency, we cannot exclude that this effect may contribute to its pathogenicity as well. Also, in a recent miRNA sequencing experiment in iPSC-derived endothelial cells from MMD patients, 41 miRNA targets involved in *STAT3-*, *IGF-1*-, and *PTEN*-signaling were shown to be downregulated in cases, suggesting an involvement of these pathways in the pathogenesis of MMA [48]. The role of *STAT3* in mediating the effects of VEGF on endothelial cells [49] and, among others, its involvement in the control of angiogenetic processes and matrix remodeling after strokes [49], provide further biological plausibility to this association. Taken together, the abovementioned information as well as the absence of additional variants in known MMA-associated genes in our patient suggest that this association may in fact be causal. Nevertheless, further clinical reports and functional studies will be needed to strengthen this finding.

In conclusion, by describing a large group of children carrying predicted deleterious *RNF213* variants we prove that this gene is associated with a more severe clinical presentation, characterized by early onset, posterior involvement and higher stroke rates in multiple territories. Because of these profound phenotypic differences, the employment of genetic testing in pediatric MMA cases could have multiple advantages. First, it would allow for the planning of tighter follow-up schedules in *RNF213*-positive children that do not present with multi-territorial involvement at diagnosis, as this is expected to appear at higher rates in this group. Second, it would allow for a cascade screening of family members, thus possibly leading to the identification of additional asymptomatic or misdiagnosed MMA cases. In the absence of evidence-based published recommendations, we would recommend performing MRI-MRA screening in asymptomatic siblings of patients with inherited *RNF213* (likely) pathogenic variants once with no further follow-up if normal. Since sedation needed may be considered too risky for asymptomatic, very young siblings, the timepoint of MRI-MRA scanning should be based on collective decision making with informed parental consent. Otherwise, except for identical twins, MRI-MRA screening is recommended in siblings of moyamoya children only if they are symptomatic [50]. A search for *RNF213* deleterious variants may provide clinical benefits when extended to MMS cases as well, as demonstrated by the two children with neurofibromatosis, *RNF213* variants and PCA involvement in our cohort. Hence, we consider comprehensive genetic testing as an indispensable tool to be employed at time of diagnosis together with brain MRIs and angiographies.

Despite the considerable achievements in the last years in unraveling the genetics of MMA, multiple areas remain open for discovery. Novel genes as well as somatic mosaicism for mutations in known MMA genes may play a significant role in its pathogenesis. Furthermore, the development of reliable functional testing for *RNF213* variants could allow for better variant classification. Finally, the penetrance of genetic risk variants should be tested in prospective cohorts.

## Supplementary information

Supplemental Figure S1

Table S1

Table S2

Table S3

Table S4

Table S5

Legend to Supplemental Figure S1

## Data Availability

Protocols and code presented in this work are available upon request to the corresponding authors. Sequence and copy number variants have been deposited in ClinVar (https://www.ncbi.nlm.nih.gov/clinvar/; submission ID SUB11907061) and Decipher (https://www.deciphergenomics.org/; see Decipher-IDs in Table S2).
