# Supplementary figures and images for: The genetic landscape and clinical implication of pediatric Moyamoya angiopathy in an international cohort
Source: Eur J Hum Genet. 2023 Apr 4;31(7):784–92. doi: 10.1038/s41431-023-01320-0 (PMC10325976; doi:10.1038/s41431-023-01320-0)

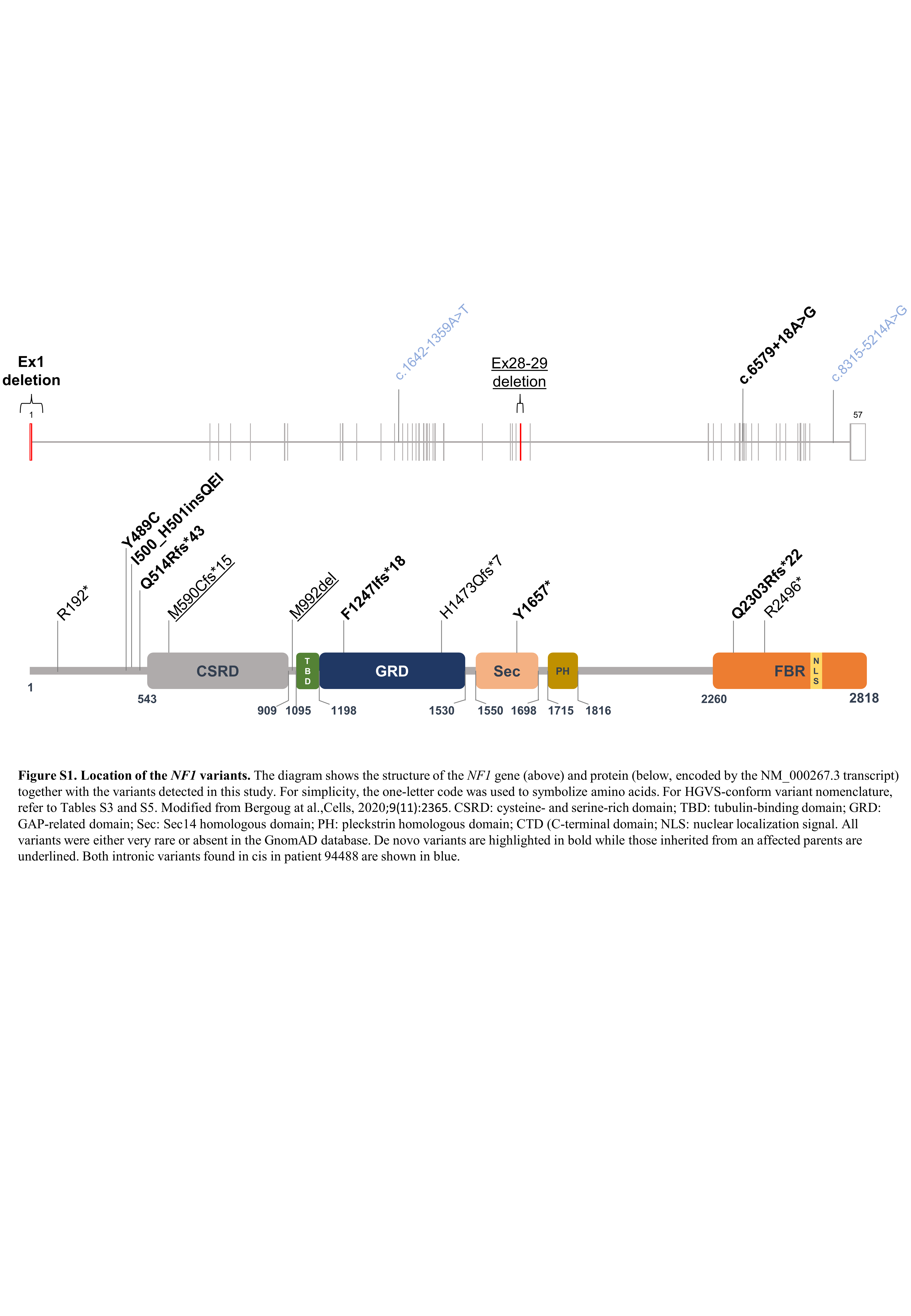

Supplement: Supplementary file 1 — Supplemental Figure S1 [file 41431_2023_1320_MOESM1_ESM.tif]
