# Supplementary material for: The genetic landscape and clinical implication of pediatric Moyamoya angiopathy in an international cohort
Source: Eur J Hum Genet. 2023 Apr 4;31(7):784–92. doi: 10.1038/s41431-023-01320-0 (PMC10325976; doi:10.1038/s41431-023-01320-0)
Supplement: Supplementary file 2 — Table S1 [file 41431_2023_1320_MOESM2_ESM.pdf]

Table S1. List of genes included in our Moyamoya WES filter and associated relevant literature

Gene ID	PMID(s)	Notes
MTHFR	29273593	GWAS hit gene
PLOD1	29273593	GWAS hit gene
TSC22D2	29273593	GWAS hit gene
HDAC9	29273593	GWAS hit gene
JAZF1	29273593	GWAS hit gene
FADS1	29273593	GWAS hit gene
FADS2	29273593	GWAS hit gene
SBF2	29273593	GWAS hit gene
LRP1	29273593	GWAS hit gene
CARD14	29273593	GWAS hit gene
RNF213	29273593	GWAS hit gene
RPTOR	29273593	GWAS hit gene
CTAGE1	29273593	GWAS hit gene
SPTLC3	29273593	GWAS hit gene
HORMAD2	29273593	GWAS hit gene
TCN2	29273593	GWAS hit gene
CBL	25283271, 28343148, 28589114, 32637631	MMA associated with Noonan phenotype and juvenile myelomonocytic leukaemia
ACTA2	19409525, 20970362, 20734336, 30300893	Arteriopathy with partial overlap with MMA; assoaciated with coronary artery disease, thoracic aortic disease, and generalized smooth muscle cell dysfunction
SAMHD1	21402907, 29869477, 20653736	MM-like arteriopathy associated with Aicardi-Guitieres syndrome (AR)
CD59	29843966	MMA associated with hemolytic anemia, peripheral neuropathy, and strokes (AR)
MYH11	29263223	Moyamoya-like cerebrovascular disease
MTFMT	27393152	Gene associated with Mitochondrial complext 1 deficiency (AR). Reported in one child with ESRD and MMA
CCER2	27717682	Recently identified candidate MMD gene
SLC7A7	27321952	Gene associated with lysinuric protein intolerance (AR). Reported once in association with MMA
ELN	27080061, 22102960	Variants reported in association with MMA and artery tortuosity. MMA was found in Williams syndrome patients as well
GUCY1A3	26777256	Gene responsible for MMA with achalasia and hypertension (AR)
C19orf45	26530418	rs140134109 enriched in Asian MMD cases in 26530418
ZNF783	26530418	rs146586179 enriched in Asian MMD cases in 26530418
MLLT6	26530418	rs2241012 enriched in Asian MMD cases in 26530418
APOA1	26530418	rs12718465 enriched in Asian MMD cases in 26530418
ZXDC	26530418	rs16837497 enriched in Caucasian and non-RNF-p.R4810K MMD cases in 26530418
RPTN	26530418	rs143744326 enriched in Caucasian and non-RNF-p.R4810K MMD cases in 26530418
CD46	26530418	rs35366573 enriched in Caucasian and non-RNF-p.R4810K MMD cases in 26530418
NR3C1	26530418	rs6195 enriched in Caucasian and non-RNF-p.R4810K MMD cases in 26530418
NAPSA	26530418	rs11670727 enriched in Caucasian and non-RNF-p.R4810K MMD cases in 26530418
PDIA4	26530418	rs2290971 enriched in non-RNF-p.R4810K MMD cases in 26530418
IL12RB2	26530418	rs2307145 enriched in non-RNF-p.R4810K MMD cases in 26530418
BRCC3	24948625, 21596366, 26691666	Found deleted either alone in MMA or together with F8 in Severe Hemophilia and Moyamoya (SHAM) syndrome
SHOC2	25858597, 25563136	MMA associated with Noonan Syndrome with Loose Anagen Hair
KRIT1	25413039	Found mutated in patient with MMA and cerebral cavernous malformations
C1QB	23651859	Found mutated in patient with SLE, progressive encephalopathy, intracranial calcification and MMA-like vasculopathy
GLA	23537685	Patients with Fabry's disease had been reported as misdiagnosed with MMA
NF1	19297575	Neurofibromatosis type 1
ANO1	Pinard et. al., Stroke;2020	Poster presentation "Missense Pathogenic Variants in ANO1 Predispose to MMD"
CHD4	31474762	ID gene; MMA belongs to clinical spectrums in de novo variant carriers
CNOT3	31474762	ID gene; MMA belongs to clinical spectrums in de novo variant carriers
SETD5	31474762	ID gene; MMA belongs to clinical spectrums in de novo variant carriers
PTPN11	21763956	MMA described in association with Noonan syndrome
HBB	11964276, 15158221	Sickle-cell disease
JAG1	29362841, 22759690, 25465847	Alagille Syndrome
SMARCAL1	9674900, http://dx.doi.org/10.7196/SAJCH.2019.v13i3.1636	Schimke immuno-osseous dysplasia and Moyamoya
NF2	30666475	Neurofibromatosis type 2
NOTCH2	29362841, 22759690, 25465847	Alagille Syndrome
IGF1R	22693602	Het. Variant in child with MOPD2 (PCNT) and MMA
PCNT	15368497, 19839044, 22693602	MOPD2
ADAMTS13	22378676, 18481107	Thrombotic thrombocytopoenic purpura
PKLR	17621533	Piruvate kinase deficiency
TGIF	16475235	Holoprosencephaly
ABCC6	16086762	PXE
F5	16009774, 32462312	F5 Leiden associated with MMA
F8	24948625, 21596366, 26691666	Deleted together with BRCC3 in SHAM syndrome
FANCC	18772355	Fanconi and MMA
SGCE	17394247	Myoclonic dystonia and MMA
EPB42	21147392, 11483402, 28221268	Spherocytosis
SLC4A1	21147392, 11483402, 28221268	Spherocytosis
SPTA1	21147392, 11483402, 28221268	Spherocytosis
SPTB	21147392, 11483402, 28221268	Spherocytosis
ANK1	21147392, 11483402, 28221268	Spherocytosis
PALD1	30908154	Novel candidate MMA gene identified in multigenerational families
DOCK8	22968740	MMA described in one patient with DOCK8 deficiency
PHACTR1	32411507	p.V265L variant associated with disease progression in MMA
GNPTAB	24878482	MMS associated with Mucolipidosis-II
MTCP1	21596366	Deleted together with BRCC3 in SHAM syndrome
MTCP1NB	21596366	Deleted together with BRCC3 in SHAM syndrome
CMC4	21596366	Deleted together with BRCC3 in SHAM syndrome
FUNDC2	21596366	Deleted together with BRCC3 in SHAM syndrome
DNAH5	32847546	Moyamoya in patient with Kartagener syndrome
DNAH11	32847546	Moyamoya in patient with Kartagener syndrome
MAGEA8	31924698	Xq28 copy number gain associated to Moyamoya
CXORF40B	31924698	Xq28 copy number gain associated to Moyamoya
MAMLD1	31924698	Xq28 copy number gain associated to Moyamoya
SPRED1	33078527	Moyamoya in patient with Legius syndrome
AFF4	33248856	Moyamoya in patient with CHOPS syndrome
CLN6	33024953	Moyamoya and epilepsy in patients with CLN6 biallelic mutations
MYH9	32950057	Moyamoya-like vasculopathy associated to MYH9-related thrombocytopenia
DIAPH1	34125151	Candidate gene for Moyamoya in non-East Asian patients
STAT3	31130284	Truncating variant found in patient with MMA
