# Supplementary material for: The genetic landscape and clinical implication of pediatric Moyamoya angiopathy in an international cohort
Source: Eur J Hum Genet. 2023 Apr 4;31(7):784–92. doi: 10.1038/s41431-023-01320-0 (PMC10325976; doi:10.1038/s41431-023-01320-0)
Supplement: Supplementary file 3 — Table S2 [file 41431_2023_1320_MOESM3_ESM.pdf]

Table S2. Results of CMA analysis. (Possibly) pathogenic CNVs are highlighted in bold.
FMD = fibromuscular dysplasia
NF1 = Neurofibromatosis type 1
DD/ID= developmental delay / intellectual disability
MOPD2 = Microcephalic osteodysplastic primordial dwarfism type II
MMD = Moyamoya disease
n.a.=not available

Patient ID	Decipher ID	Sex	MMD / Syndromic features	CMA results	Inheritance / Comments
76572	n.a.	f	Vertebral artery stenosis, possible FMD	negative	
84980	n.a.	f	D	negative	
68632	n.a.	f	NF1	negative	
69035	453986	f	NF1	185 Kb dup chr1:109515027-109700157, genes: WDR47, TAF13, TMEM167B, SCARNA2, C1orf194, KIAA1324	Duplication reported once in patient with obesity and poor coordination (Decipher ID305835). Overlapping duplications found in control populations (Affy6)
69946	453987	f	Bilateral optic nerve colobomas, congenital nystsagmus, normal development, has twin sister wout moyamoya	237 Kb dup chr15:22845175-23082328, genes: TUBGCP5, CYFIP1, NIPA2, NIPA1	In a large study on the effect of CNVs on cognition, 136 control individuals carrying the 15q11.2 duplication performed to a similar level as population controls on all tests of cognitive function (PMID: 24352232)
70160	453988	m	Unilateral renal artery stenosis	92 Kb del chr5: 108974885-109066850: genes: MAN2A1	Not previously reported in Decipher; no dose sensitive genes involved; MAN2A1 is GWAS hit for schizophrenia and intelligence (PMID: 32377000)
70296	454732	f	D	37 Kb dup chrX:137660905-137697444; gene: FGF13	Not previously reported in Decipher; lies at the edge of the FGF13 gene
70863	414777	m	D	30 Kb dup chr5:112334856-112364899, genes: DCP2, MCC	Not previously reported in Decipher; inherited from healthy father
71070	435911	m	D	119 Kb dup chr8:144972667-145091835, Gene: PLEC, MIR661, PARP10, GRINA und SPATC	No similar duplications in Decipher
71131	414780	f	D	58 Kb del chr20:60021785-60079599, gene: CDH4	The same or very similar deletions reported in Decipher as likely benign or inherited from normal parent. Not present in the mother. Father could not be tested.
71191	414781	m	D	A. Possible 20.19 kb del, chr4:85852467-85872655, intragenic in WDFY3 B. 79kb del, chr19:43846269-43925290, genes. TEX101, CD177, CD177P1	A. Not present in Deciper. The deletion affects one non-coding exon. Phenotype incompatible with WDFY3 LOF (macrocephaly, ID, PMID: 31327001). B. The same as well as overlapping deletions were reported in control populations (e.g. GnomAD: DEL_19_172641).
71248	453989	f	D	69 Kb dup chr2:130805310-130874475; genes: LOC440905, POTEF	Overlapping duplications found in control populations (6x in DDD; GnomAD: DUP_2_6068, DUP_2_6076)
71819	n.a.	f	FMD	negative	
72299	414786	m	D	30.3 Kb del in chr2:62197877-62228180, intragenic in COMMD1	Same or very similar deletions reported as recurrent variants in multiple populations (Affy6, DDD)
72370	453990	m	Trisomy 21	A. **Trisomy 21**; B. 138 Kb dup chr11:61943817-62081769; genes: SCGB1D1, SCGB2A1, SCGB1D2, SCGB2A2, SCGB1D4	B. Not previously reported in Decipher. Smaller overlapping deletions seen in control populations (GnomAD DUP_11_32568)
72638	453991	m	D, familial case	96 Kb dup chr13 hg19: 113417472-113513306; genes: ATP11A	Not previously reported in Decipher. Multiple smaller duplications in this region reported in control populations (GnomAD structural variants)
73022	n.a.	f	D	negative	
73297	487182	f	D	A. 93 Kb dup in chr3:195585686-195678475, gene: TNK2<br>B. 77 Kb del in chr16:6405941-6482782, gene: RBFOX1<br>C. 43 Kb dup in chr1:152611169-152653702, genes: LCE6A and SMCP	A. inherited from healthy mother. B and C. inherited from healthy father.
73577	414789	f	Mid-aortic syndrome, fructose malabsorption	170 Kb dup in chr13:71967546-72137253, gene: DACH1	Inherited from healthy mother. Slightly smaller duplication reported in Decipher as likely benign (ID288092)
73732	414790	f	ID/DD, dysmorphic features	A. **598 Kb heterozygote deletion in chr16:29580020-30178406. Band 16p11.2**. B: **2.1 Mb copy number gain (4 copies) in chr1: 145895746-148020154**	A. Recurrent pathogenic 16p11.2 deletion. B. Recurrently duplicated region in 1q21.1-21.2. Duplications have been reported in control populations (Affy6, GDV: gssvG1845). Triplications described in association with dysmorphic features, increased weight and macrocephaly (PMID: 26327614).
73955	414917	f	D	A. Possible 38kb dup in chr20:2599120-2636857, genes: TMC2, NOP56, MIR1292, SNORD110, SNORA51 and SNORD86. B. Possible 30kb del in chr17:29854791-29884676, genes: RAB11FIP4 and MIR4724.	A. not present in Decipher. B. Not present in Decipher. RAB11FIP4 is the gene besides NF1, but clinically not NF1
74316	414920	f	Spherocytosis	437 Kb dup in chr7:56189912-56627061, genes: CCNJP1, CICP8, NMD3P2, RBM22P3, RNU6-1052P, RNU6-1335P, VN1R25P	No protein-coding genes involved. Smaller, partially overlapping duplications seen in control populations (Affy6)
74480	435909	f	Dysmorphic features, no ID before onset of MMA, FV-Leiden heterozygote	97kb duplication chr4:3225613-3322401, gene: HTT, MSANTD1, RGS12	No similar duplications in Decipher
74567	414922	m	NF1	A. 1.28 Mb dup in chr4:127859556-129143362, genes: INTU, SLC25A31, HSPA4L, PLK4, MFSD8, C4orf29, LARP1<br>B. 86 Kb dup in chr9:93599546-93684714, Gen: SYK	A. De novo; bands 4q28.1 to q28.2; Not present in Decipher. B. Seen 2x in Affy6 control population; inherited from healthy father
74911	n.a.	f	D	negative	
74962	414915	m	NF1	341 Kb dup in chr1:240807305-241148036, gene: RGS7	Inherited from healthy mother. Not present in Decipher; overlapping dup 1x in Affy6 control population
75129	414924	f	D	237 Kb dup in chr1:58601402-58838701, gene: DAB1	Not present in the mother. Father not tested. A slightly larger, overlapping duplication is described in Decipher as inherited from normal parent
75691	n.a.	$	Spherocytosis	negative	
75797	414925	f	D	A. 35 Kb dup in chr3:8779633-8814696, genes: CAV3, OXTR, SSUH2	Inherited from healthy father
76421	414927	f	D	A. 25 Kb dup in chr3:184003967-184028850, genes: ECE2, PSMD2	Inherited from healthy mother
76654	414704	f	Hypoplastic bulbus oculi, unilateral; no DD/ID	**835 Kb del in chr1:10544987-11380118, genes: PEX14, CASZ1, C1orf127, TARDBP, MASP2, SRM, EXOSC10, MTOR, MTOR-AS1, ANGPTL7, UBIAD1; region: 1p36.22**	Maternally inherited. Not present in control populations. Overlapping comparable deletions seen in patients with developmental disorders (Decipher ID 325801 and 402363). The mother has arrhythmia. CASZ1 truncating variants have been associated with dilatative CM as well as LVNC-CM, plus CASZ1 is a tumor suppressor gene that is often deleted in brain tumors (sister of patient died at 9 of pontine glioma)
76728	414685	m	Severe hemophilia, dysmorphic features	A. **209 Kb del in chrX:154120620-154329983, genes: F8, FUNDC2, MTCP1NB, MTCP1, BRCC3**. B. 112 Kb dup in chrX:154008302-154120600, genes: MPP1, CXorf68, F8, H2AFB3, H2AFB1, F8A1, F8A3, F8A2, MIR1184-3, MIR1184-2, MIR1184-1. C.<br>148 Kb dup in chrX:38486799-38634614, gene: TSPAN7.	A. Deletions of F8 and BRCC3 are associated with SHAM syndrome (severe hemophilia and Moyamoya; PMID: 24948625, 21596366). B. This duplication lies just beside the deletion in A and could play a modulating role in the phenotype; it's not part of the classical SHAM deletion. The rearrangement on the X chromosome is inherited from the healthy mother. C. Seen already in Affy control population
76912	n.a.	f	D	negative	
77643	414930	f	D	83 Kb dup in chr2:242045078-242127775, genes: PASK, PPP1R7	Inherited from healthy father.
78168	n.a.	m	D	negative	
78165	n.a.	m	Dysmorphic features, no ID before onset of MMA, FV-Leiden heterozygote	negative	
78595	414931	m	Morning glory anomaly of right eye	165 Kb del in chr4:8202210-8367354, genes: SH3TC1, HTRA3	Not present in the mother. Father not tested. Overlapping deletions in this region were reported as benign in Decipher and in the ISCA population as well. No dosis-sentivie genes involved
78715	487186	f	Trisomy 21	A. **Trisomy 21**. B. 232 Kb dup in chr9:118965960-119198325, genes: PAPPA, ASTN2. C. 117 Kb dup in chrX:69207742-69324543, genes: EDA, MIR676, AWAT2, OTUD6A. D.<br>31 Kb deletion in chr6:153028369-153059730, gene: MYCT1.	B. Inherited from healthy father. C and D. Inherited from healthy mother
79502	414956	f	NF1	A. 165 Kb del in chr13:101140308-101305382, genes: PCCA, A2LD1, TMTC4. B. Posisble 24 Kb homozygote del in chr19:51321390-51345568, genes: MGC45922, KLK1, KLK15.	A. inherited from healthy mother. B. Possibly de novo. The locus hosts a variety of recurrent CNVs: PMID 23894413. KLK15 and KLK1 are not associated to any disease in OMIM
79663	414957	m	D	A. 47 Kb dup in chr19:42259135-42305800, genes: CEACAM6, CEACAM3	A. Not present in the mother. Father could not be tested. A similar duplication was reported in the Affy6 control population.
80020	414958	f	D	73 Kb del in chr19:22245897-22319312, gene: ZNF257	Already reported 2x as benign in ISCA
80692	414960	f	D	A. 218 Kb dup in chr22:43046354-43264400, genes: A4GALT, ARFGAP3. B. 64 Kb dup in chrX:152549732-152613590, gene: ZNF275.	A. Inherited from healthy father. Overlapping duplication present in the Affy6 control population. B. Inherited from healthy mother.
80819	n.a.	f	NF1	negative	
82059	n.a.	f	NF1	negative	
83200	n.a.	m	NF1	negative	
83661	414962	f	D	262 Kb dup in chr5:102878143-103139826, gene: NUDT12	Much larger duplications in the same region reported in Decipher as inherited from normal parent
83831	414963	f	FMD, autoimmune manifestations, livedo racemosa	21 Kb del in chr5:55201090-55221769 IL31RA (cutaneous amyloidosis, dominant)	Not present in Decipher; deletion lying downstream of IL6ST (AR HyperIgE Syndrome). Exome sequencing did not reveal relevant variants in IL31RA or IL6ST that could suggest a recessive inheritance.
83955	n.a.	m	Language delay	negative	
84522	n.a.	m	NF1	negative	
85069	n.a.	f	ACTA2	negative	
84982	414964	m	Alagille	146 Kb dup in chr17:48829575-48975595, genes: LUC7L3, LINC00483, WFIKKN2, TOB1, LOC400604	Not present in Decipher/ISCA. No disease-associated genes involved
86942	n.a.	f	D	negative	
87022	414966	f	D	116 Kb del in chr13:48661549-48777222, gene: MED4	Inherited from healthy father. Deletions involving MED4 are described as benign and parentally inherited in Decipher (ID 278279)
87663	n.a.	m	D	negative	
87654	414969	m	Short stature, DD (possibly secundary to MMA)	A. 193 Kb dup in chr20: 24584177-24777177, gene: SYNDIG1 B. 144 Kb del in chr11:85403254-85547328, gene: SYTL2	A. Not present in Decipher/ISCA. No disease-associated genes involved. B. Deletions within SYTL2 observed 29x in the Affy6 control population
87885	n.a.	f	D	negative	
88103	n.a.	f	NF1	negative	
88905	414970	m	MOPD2	A. 238 Kb del in chr12:83340298-83577832, gene: TMTC2 B. 212 Kb dup in chr13:19853294-20065373, genes: ANKRD26P3, LINC00421, TPTE2 C.114 Kb del in chr4:71167862-71281554, genes: CABS1, SMR3A, SMR3B, PROL1. D. 24 Kb dup in chr21:15909250-15933011, gene: SAMSN1.	A. Disrupts TMTC2 (not dosis-sentivie gene). No disease-associated genes involved. B. Reported as likely benign in ISCA (nssv1609016) C. Recurrent, likely benign deletion. D. Similar SAMSN1 intragenic dup reported 1x in Affy6 control population
88884	n.a.	f	NF1	negative	
89165	n.a.	f	D	negative	
90045	n.a.	m	D	negative	
90048	415021	f	D	676 Kb dup in chr10:56013088-56689571, gene: PCDH15	Inherited from healthy mother. Intragenic PCDH15 duplication. Rearrangements involving this gene are responsible for Usher syndrome (AR)
90549	n.a.	m	Aortic Isthmus stenosis	negative	
91071	387235	m	D	Duplikation 467kb chrX: 91570172-92037656; Gene: PCDH11X	Maternally inherited; overlapping deletion have been reported as likely benign in Decipher (ID 288548) and have been reported in control populations (DGV Gold, GnomAD structural variants)
90101	n.a.	m	D	negative	
92357	n.a.	f	D	negative	
93045	391536	f	ACTA2	1.1Mb dup chr2: 159141104-160268920, Gene: CCDC148, PKP4, DAPL1, TANC1, WDSUB1, BAZ2B	Maternally inherited. Unclear interpretation based on Decipher data.Could break one dose-sensitive gene (BAZ2B).
93051	n.a.	f	D	negative	
94029	n.a.	m	D	negative	
94488	454110	m	NF1	possible 36kb del chr6:161032733-161068954; genes: LPA, intragenic	
94566	454336	f	D	38kb del chr1:65673550-65711982, Gene AK4	AK4 intragenic; AK4 is not dosis-sensitive
95246	n.a.	m	D	negative	
95540	n.a.	f	NF1	negative	
62695	454337	f	Immunodeficiency, pure red cell aplasia, pseudotumor cerebri	dup 63 Kb chr20:47715384-47777906, Gene: STAU1	Overlapping duplications found in control populations (1x in DDD; GnomAD: DUP_20_49309)
95538	487191	f	Trisomy 21, Evans syndrome	**Trisomy 21**	
96282	415022	f	D	180 Kb dup in chr2:197361533-197541555, Gene: HECW2, CCDC150	Same duplication reported 2x in the Affy6 control population
97355	415024	m	COL4A2-related brain small vessel disease type 2, poroencephalic cyst in MRI	23 Kb del in chr3:131075516-131098387, genes: LOC339874, NUDT16P1	No dose-sensitive genes involved. No similar deletion in the online databases
97764	454338	f	D	A. del 507 Kb chr1:145382123-145888926, genes: GPR89A, PDZK1, CD160, RNF115, POLR3C, RBM8A, PIAS3. B. Possible deletion of max 74 Kb chr1: 147399549-147473821, Gene: GPR89B, GPR89C, PDZK1P1.	A. Deletions in this region were reported in control populations: GnomAD DEL_1_7663; also reported multiple times as pathogenic in Clinvar and Decipher. The deletion affects a susceciptibility locus for TAR-syndrome (RBM8, AR). B. No dosis-sensitive genes involved; overlapping deletions found in control populations (GnomAD: DEL_1_7778).
98340	n.a.	m	D	negative	
98932	414953	f	NF1	55 Kb del in chr15: 100728676-100783256, Gene: ADAMTS17, intragenic	ADAMTS17 is responsible for Weill-Marchesani syndrome type 4, AR
99265	n.a.	m	D; Dysmorphic features, no DD/ID	negative	
75558	n.a.	m	Ocular apraxia, cerebellar anomalies, ataxia, DD: lower-normal range, obesity	negative	
78053	n.a.	m	D	negative	
78603	487192	m	T1DM, Hypothyroidism, GH-Deficiency, short stature, IgA-deficiency, microcephaly	94 Kb del in chr2:64089398-64183851, genes: UGP2 und VPS54	De novo. UGP2 associated with AR epileptic encephalopathy. Partially overlapping deletion affecting VPS54 only present 1x in Affy6 control population
81910	n.a.	m	D	negative	
94084	n.a.	f	NF1	negative	
100129	n.a.	m	D	negative	
100132	n.a.	f	D	negative	
