# Supplementary material for: The genetic landscape and clinical implication of pediatric Moyamoya angiopathy in an international cohort
Source: Eur J Hum Genet. 2023 Apr 4;31(7):784–92. doi: 10.1038/s41431-023-01320-0 (PMC10325976; doi:10.1038/s41431-023-01320-0)
Supplement: Supplementary file 5 — Table S4 [file 41431_2023_1320_MOESM5_ESM.pdf]

Table S4. List of variants in the *RNF213* gene in our MMA cohort. MAF: Minor allele frequency in all GnomAD exomes. PMID: PubMed ID. For the Combined prediction column: D: deleterious; PD: possibly deleterious; B: benign.
Heterozygotes for predicted deleterious and possibly deleterious variants have been labelled as "RNF213 patients" in this study, as indicated in the specific column.
Variants previously reported in individuals with MMA are highlighted in bold
*The R4810K variant was reclassified as possibly deleterious based on the available literature

Patient ID	MMD/MMS	Syndrome / additional manifestations	Variant	Parental origin	MAF	CADD	VIPUR	Combined prediction	"RNF213 patient"	Other variants in MM filter 13.08.21 (excl RNF213)	PMID for previously reported variants (PMID)
99799	D		c.12341C>A,p.(Thr4114Lys) / c.1214_1216del,p.(Gly405del)	NA / NA, both variants absent in mother	NA/NA	34/12.75	0.56/NA	**D**/B	yes	MTHFR: NM_001330358.2,c.1127G>A,p.(Arg376His), CADD27.9, Clinvar: P, Intervar: LP; NOTCH2: NM_001200001.2,c.17_18del,p.(Pro6Argfs*27), CADD: NA; F5: NM_000130.5,c.112G>T,p.(Ala38Ser), CADD: 26.8, PCNT: NM_001315529.2,c.1800G>A,p.(=), CADD: NA; PCNT: NM_001315529.2,c.4351_4353del,p.(Glu1451del), CADD: NA ; DNAH11: NM_001277115.2,c.8572G>A,p.(Gly2858Ser), CADD: 34	
82059	S	NF1	c.5114C>A,p.(Thr1705Lys)	NA, not present in mother	0.0043	32	0.88	**D**	yes	NF1: NM_000267.3,c.7486C>T,p.(Arg2496*), Clinvar:P, Intervar:P	
70863	D		c.5114C>A,p.(Thr1705Lys)	M	0.0043	32	0.88	**D**	yes	SBF2: NM_030962.4,c.4054A>G,p.(Ser1352Gly); MTFMT: NM_139242.4,c.172T>A,p.(Phe58Ile)	
73577	D	Mid-aortic syndrome	c.5114C>A,p.(Thr1705Lys) / c.3160T>A,p.(Leu1054Met)	M / M	0.0043 / NA	32 / 8.578	0.8 / 0.46	**D / PD**	yes	LRP1: NM_002332.3,c.2308C>T,p.(Arg770Trp); LRP1: NM_002332.3:c.3644G>A, p.(Gly1215Glu), CADD 25.9/24.7, compound het	
72638	D, familial case		**c.12185G>A,p.(Arg4062Gln)**	**M**	NA	31	0.69	**D**	yes	none	p.(Arg4062Gln): 21799892, 25964206, 29387438
74480	D	FVLeiden het	**c.12185G>A,p.(Arg4062Gln)** / c.6979A>G,p.(Asn2327Asp)	**M** / NA, absent in mother	NA / 0.0011	31 / 0.049	0.69 / 0.46	**D / PD**	yes	none	p.(Arg4062Gln): 21799892, 25964206, 29387438; p.(Asn2327Asp): 31170158, 32271753
78168	D		c.12374A>G,p.(Asp4125Gly)	P	NA	31	0.86	**D**	yes	NOTCH2: NM_024408.4,c.6169dup,p.(Asp2057Glyfs*8); NF1: NM_000267.3,c.7457C>T,p.(Thr2486Ile), rs149055633, ClinVar: VOUS, LOVD: benign	
73297	D		c.12374A>G,p.(Asp4125Gly)	NA	NA	31	0.86	**D**	yes	SPTA1: NM_003126.4,c.2600C>A,p.(Ala867Glu)	
94029	D		c.12040C>T,p.(His4014Tyr) / c.6979A>G,p.(Asn2327Asp) / c.14194A>G,p.(Lys4732Glu)	De novo / M / P	NA / 0.0011 / 0.0077	29.7 / 0.049 / 4.014	0.78 / 0.46 / 0.18	**D / PD** / B	yes	DIAPH1: NM_001079812.3,c.1742G>T,p.(Gly581Val); CADD: 24.5	p.(Asn2327Asp): 31170158, 32271753
80692	D		c.11998T>C,p.(Cys4000Arg)	M	NA	26.9	0.89	**D**	yes	none	
100129	D		c.11998T>C,p.(Cys4000Arg) / c.6551A>G,p.(Gln2184Arg)	De novo / P	NA / 0.0025	26.9 / 0.005	0.89 / 0.19	**D** / B	yes	PCNT: NM_001315529.2,c.4348G>A,p.(Glu1450Lys), CADD 25.7; ADAMTS13: NM_139025.5,c.559G>C,p.(Asp187His), CADD 25.6	
71070	D		c.12049T>C,p.(Cys4017Arg)	NA, absent in father	NA	26.1	0.88	**D**	yes	ABCC6: NM_001079528.4,c.133_138del,p.(Trp45_Val46del); ZNF783: NM_001195220.2,c.378G>T,p.(Trp126Cys), CADD 26.8	
71131	D		c.12059G>A,p.(Cys4020Tyr)	NA, absent in mother	NA	26	0.93	**D**	yes	DNAH5: NM_001369.3,c.6049C>T,p.(Arg2017Trp), CADD: 29.4	
98340	D		c.12345A>C,p.(Lys4115Asn)	M	NA	25.1	0.84	**D**	yes	SPTB: NM_000347.5,c.5926G>A,p.(Ala1976Thr), CADD34	
84980	D		c.14546T>A,p.(Ile4849Asn)	M	NA	24.9	0.46	**D**	yes	none	
78053	D		c.13913C>T,p.(Thr4638Ile)	NA	0.0007	24.6	0.67	**D**	yes	none	
90101	D		c.9668C>T,p.(Ser3223Leu)	M	8.13E-06	34	0.26	**PD**	yes	none	
74316	S	Spherocytosis	**c.12055C>T,p.(Arg4019Cys)**	**M**	0.0006	24	0.26	**PD**	yes	ANK1: NM_000037.4,c.4140del,p.(Leu1382Trpfs*24); LRP1: NM_002332.3,c.7636G>A,p.(Gly2546Ser), CADD 27.3	p.(Arg4019Cys): 25278557, 27736983
68632	S	NF1	**c.11779G>A,p.(Ala3927Thr)**	**P**	4.06E-06	14.51	0.499	**PD**	yes	NF1: NM_000267.3,c.1502_1503insGGAAATTCA,p.(Ile500_His501insGlnGluIle), Clinvar: unknown, Intervar: LP; SBF2: NM_030962.4,c.3824G>A,p.(Arg1275His), SPTB: NM_000347.5,c.4075C>T,p.(Arg1359Trp)	p.(Ala3927Thr): 30908154
91071	D		**c.14429G>A,p.(Arg4810Lys)** / c.14194A>G,p.(Lys4732Glu)	**M / P**	0.0003 / 0.0077	7.374 / 4.014	0.2 / 0.18	**PD*** / B	yes	CD46: NM_002389.4,c.811_816del,p.(Asp271_Ser272del); SLC4A1: NM_000342.4,c.2116C>A,p.(Leu706Met), CADD: 25.9; NOTCH2: NM_001200001.2,c.17_18del,p.(Pro6Argfs*27), CADD: NA	p.(Arg4810Lys): 26530418
76912	D		c.8212C>G,p.(Leu2738Val)	P	2.04E-05	14.42	0.16	B	no	none	
79502	S	NF1	**c.11884A>G,p.(Asn3962Asp)**	**P**	4.47E-05	13.17	0.1	B	no	none	p.(Asn3962Asp): 21799892
95246	D		c.14194A>G,p.(Lys4732Glu)	P	0.0077	4.014	0.18	B	no	DNAH5: NM_001369.3,c.1126G>T,p.(Ala376Ser), CADD: 24.7	
85069	S	ACTA2	c.14194A>G,p.(Lys4732Glu)	P	0.0077	4.014	0.18	B	no	ACTA2: NM_001141945.3,c.536G>A,p.(Arg179His), CADD 25.1, Clinvar: P, Intervar: VUS	
78603	S	T1DM, Hypothyroidism, GH-Deficiency, short stature, IgA-deficiency, microcephaly	c.6350G>A,p.(Arg2117His)	NA	1.63E-05	2.743	0.1	B	no	SLC7A7: NM_001126105.3,c.1382_1384del,p.(Ile461del); MYH9: NM_002473.6,c.4298G>A,p.(Arg1433His), CADD: 32	
69035	S	NF1	c.13250G>A,p.(Arg4417His)	NA	0.0006	0.088	0.06	B	no	NF1: NM_000267.3,c.4419_4420del,p.(His1473Glnfs*7); LRP1: NM_002332.3,c.7636G>A,p.(Gly2546Ser); MTFMT: NM_139242.4,c.188T>C,p.(Leu63Pro)	
87654	S	Short stature, developmental delay	c.9952A>G,p.(Ile3318Val)	P	0.0025	0.06	0.09	B	no	none	
