# Supplementary material for: The genetic landscape and clinical implication of pediatric Moyamoya angiopathy in an international cohort
Source: Eur J Hum Genet. 2023 Apr 4;31(7):784–92. doi: 10.1038/s41431-023-01320-0 (PMC10325976; doi:10.1038/s41431-023-01320-0)
Supplement: Supplementary file 6 — Table S5 [file 41431_2023_1320_MOESM6_ESM.pdf]

Table S5. List of variants in the *NF1* gene in our MMA cohort. All variants are annotated based on the NM 000267.3 transcript. All variants were detected in heterozygosity. †=Intronic variants detected by whole genome sequencing.
Variant pathogenicity was evaluated according to ACMG guidelines (Richards et al., Genet Med. 2015;17(5):405-424)

Patient ID	Clinical presentation	Age at diagnosis (months)	Clinical presentation	NF1 Variant	MLPA	Conv. Karyotyping	Parental origin	Notes	ACMG	Clinvar	LOVD
**68632**	NF1	16	Stroke, focal seizures	c.1502_1503insGGAAATTCA,p.(Ile500_His501insGlnGluIle)	NA	NA	De novo	not in GnomAD	Likely pathogenic (II), PS2, PM2, PM4, PP4		Not present.2 missense variants (1 VUS-pathogenic, 1 pathogenic) at c.1496
**69035**	NF1	36	TIA	c.4419_4420del,p.(His1473Glnfs*7)	NA	NA	NA	not in GNomAD	Pathogenic (Ic), PVS1, PM2, PP4		Not present. c.4415del pathogenic
**82059**	NF1	43	MRI for underlying disease	c.7486C>T,p.(Arg2496*)	NA	NA	NA	rs866445127, 1x in GnomAD	Pathogenic (Ia), PVS1, PP4, PS4	pathogenic	pathogenic
**83200**	NF1	72	Migraine	none	Deletion of Ex 28 and 29	NA	Inherited from affected father		Pathogenic (Id), PVS1, PP1, PP4		
**74567**	NF1	16	TIA	c.1466A>G,p.(Tyr489Cys)	NA	NA	De novo	rs137854557, 3x in GnomAD	Pathogenic (II), PS2, PS4, PP4	pathogenic	pathogenic. "splicing affected"
**74962**	NF1	150	MRI for underlying disease	c.1768del,p.(Met590Cysfs*15)	NA	NA	Inherited from affected mother	not in GNomAD	Pathogenic (Ic), PVS1, PM2, PP4		not present
**79502**	NF1	14	Stroke, hemiparesis	none	Deletion of Ex1	NA	De novo		Pathogenic (Ia), PVS1, PS2, PP4		
**84522**	NF1	43	MRI for underlying disease	c.1541_1542del,p.(Gln514Argfs*43)	NA	NA	De novo	rs267606600, not in GnomAD	Pathogenic (Ia), PVS1, PS2, PM2, PP4, PP5	pathogenic	pathogenic
**80819**	NF1	98	Stroke, hemiparesis	c.2970_2972del,p.(Met992del)	NA	NA	Inherited from affected mother	rs267606606, not in GnomAD	Likely pathogenic (II), PS4, PM2, PM4, PP4	pathogenic	pathogenic
**88103**	NF1	100	Tiredeness, school issues	c.574C>T,p.(Arg192*)	NA	NA	NA	rs397514641, MAF GnomAD 0.000003990	Pathogenic (Ia), PVS1, PS4, PP4	pathogenic	pathogenic
**88884**	NF1	13	Stroke, hemiparesis	c.4971_4977del,p.(Tyr1657*)	NA	NA	De novo	not in GNomAD	Pathogenic (Ia), PVS1, PS2, PM2, PP4		Not present. c.4972-4973 pathogenic
**98932**	NF1	71	Stroke	c.3739_3742del,p.(Phe1247Ilefs*18)	NA	NA	De novo	not in GNomAD / Clinvar	Pathogenic (Ia), PVS1, PS2, PM2, PP4, PP5		pathogenic
**94488**	NF1	49	MRI for underlying disease	c.1642-1359A>T†; c.8315-5214A>G†	negative	negative	Both variants inherited from apparently not affected mother	rs531397169, MAF GnomAD: 0.00009563 / rs180775394, MAF GnomAD: 0.0008279	Likely benign (I), BS2, BP4 / VUS, BS2	not present / not present	not present / not present
**95540**	NF1	115	Headache	c.6579+18A>G†	negative	NA	De novo	Published functional studies demonstrate a damaging effect: abnormal splicing (Sabbagh et al., 2013;; PMID: 23913538). Not in GnomAD	Pathogenic (II), PS2, PS4, PS3, PM2, PP4	2x likely pathogenic; 2x VOUS	pathogenic
**94084**	NF1	109	Headache	c.6908del,p.(Gln2303Argfs*22)	NA	NA	De novo	not in GNomAD	Pathogenic (II), PS2, PS4, PS3, PM2, PP4, PP3	not reported	Not present. p.Gln2303* pathogenic
**78168**	MMD	13	TIA	c.7457C>T,p.(Thr2486Ile)	NA	NA	Maternal	rs149055633; MAF GnomAD: 0.00005567	Likely benign (II), BP4, BP6	VOUS	benign
**78165**	FVLeiden het, Dysmorphic features	10	Stroke, hemiparesis	c.7532C>T,p.(Ala2511Val)	NA	NA	Paternal	rs148154172; MAF GnomAD: 0.0007365	VUS; BP6	benign/likely benign	likely benign
