# Supplementary material for: The genetic landscape and clinical implication of pediatric Moyamoya angiopathy in an international cohort
Source: Eur J Hum Genet. 2023 Apr 4;31(7):784–92. doi: 10.1038/s41431-023-01320-0 (PMC10325976; doi:10.1038/s41431-023-01320-0)
Supplement: Supplementary file 7 — Legend to Supplemental Figure S1 [file 41431_2023_1320_MOESM7_ESM.docx]

**Figure S1. Location of the *NF1* variants.** The diagram shows the structure of the *NF1* gene (above) and protein (below, encoded by the NM_000267.3 transcript) together with the variants detected in this study. For simplicity, the one-letter code was used to symbolize amino acids. For HGVS-conform variant nomenclature, refer to Tables S3 and S5. Modified from Bergoug at al.,Cells, 2020;9(11):2365. CSRD: cysteine- and serine-rich domain; TBD: tubulin-binding domain; GRD: GAP-related domain; Sec: Sec14 homologous domain; PH: pleckstrin homologous domain; CTD (C-terminal domain; NLS: nuclear localization signal. All variants were either very rare or absent in the GnomAD database. De novo variants are highlighted in bold while those inherited from an affected parents are underlined. Both intronic variants found in cis in patient 94488 are shown in blue.
