# Supplementary material for: The genetic landscape and clinical implication of pediatric Moyamoya angiopathy in an international cohort
Source: Eur J Hum Genet. 2023 Apr 4;31(7):784–92. doi: 10.1038/s41431-023-01320-0 (PMC10325976; doi:10.1038/s41431-023-01320-0)
Supplement: Supplementary file 4 — Table S3 [file 41431_2023_1320_MOESM4_ESM.pdf]

Table S3. List of variants found in our Moyamoya WES filter. All variants in NF1,and RNF213 as well as all variants with CADD scores above 24 are shown. All variants were heterozygous except when indicated. †=variants detected by whole genome sequencing
P: pathogenic, LP: likely pathogenic, VUS: uncertain significance
P/LP variants according to either Clinvar or Intervar underlined

Patient ID	Sex	MMD/MMS	Clinical presentation	MM filter (All RNF + NF1 + CADD>24)	Notes/Refs	Phenotype-specific variants (PSVs)	ACMG evalutaion of PSVs	Additional filter	Additional filter variants	Additional pathogenic CNV (see Table S2)
62695	f	S	STAT3-related Immunodeficiency, Pure Red Cell Aplasia, pseudotumor cerebri	<u>STAT3: NM_003150.4,c.2141C>T,p.(Pro714Leu), CADD: 24.9, Clinvar: P/LP, Intervar: LP</u>	STAT3 p.Pro714Leu variant characterized in Mauracher et al., J Allergy Clin Immunol. 2020;145(4):1297-1301	STAT3: NM_003150.4,c.2141C>T,p.(Pro714Leu)	Pathogenic (II), PS3, PS4, PP3, PP4			
68632	f	S	NF1	<u>NF1: NM_000267.3,c.1502_1503insGGAAATTCA,p.(Ile500_His501insGlnGluIle), Clinvar: unknown, Intervar: LP</u>; SBF2: NM_030962.4,c.3824G>A,p.(Arg1275His), SPTB: NM_000347.5,c.4075C>T,p.(Arg1359Trp), RNF213: NM_001256071.3,c.11779G>A,p.(Ala3927Thr)	See Table S5; see Table S4	NF1: NM_000267.3,c.1502_1503insGGAAATTCA, p.(Ile500_His501insGlnGluIle)	Likely pathogenic (II), PS2, PM2, PM4, PP4			
69035	f	S	NF1	NF1: NM_000267.3,c.4419_4420del,p.(His1473Glnfs*7); LRP1: NM_002332.3,c.7636G>A,p.(Gly2546Ser); MTFMT: NM_139242.4,c.188T>C,p.(Leu63Pro); RNF213: NM_001256071.3,c.13250G>A,p.(Arg4417His)	See Table S5; see Table S4	NF1: NM_000267.3,c.4419_4420del,p.(His1473Glnfs*7)	Pathogenic (Ic), PVS1, PM2, PP4			
69946	f	D	Bilateral optic nerve coloboma, congenital nystagmus, no DD; twin sister wout moyamoya	nothing relevant						
70160	m	D	Unilateral renal artery stenosis	SPTB: NM_000347.5,c.5527C>T,p.(Arg1843Trp); PDIA4: NM_004911.5,c.350T>C,p.(Val117Ala)						
70296	f	D	D	PCNT: NM_006031.6,c.6218T>C,p.(Leu2073Pro)						
70863	m	D	D	RNF213: NM_001256071.3,c.5114C>A,p.(Thr1705Lys), CADD 32; SBF2: NM_030962.4,c.4054A>G,p.(Ser1352Gly); MTFMT: NM_139242.4,c.172T>A,p.(Phe58Ile)	See Table S4					
71070	m	D	D	RNF213: NM_001256071.3,c.12049T>C,p.(Cys4017Arg), CADD 26.1; ABCC6: NM_001079528.4,c.133_138del,p.(Trp45_Val46del); ZNF783: NM_001195220.2,c.378G>T,p.(Trp126Cys), CADD 26.8	See Table S4					
71131	f	D	D	<u>RNF213: NM_001256071.3,c.12059G>A,p.(Cys4020Tyr), CADD 26, Clinvar: unknown, Intervar: LP</u>; DNAH5: NM_001369.3,c.6049C>T,p.(Arg2017Trp), CADD: 29.4	See Table S4					
71191	m	D	D	SPTA1: NM_003126.4,c.1982G>A,p.(Arg661His); PCNT: NM_001315529.2,c.8333T>C,p.(Leu2778Pro); ANO1: NM_018043.7,c.2140G>A,p.(Glu714Lys), CADD 24.8						
71248	f	D	D	ABCC6: NM_001171.6,c.1699G>A,p.(Ala567Thr)						
71819	f	D	D	GUCY1A1: NM_001130684.3,c.1422del,p.(Lys475Argfs*60), <u>homo</u>	GUCY1A3 mutations are associated with Moyamoya 6 with achalasia, AR, OMIM 615750	GGUCY1A1: NM_001130684.3,c.1422del,p.(Lys475Argfs*60), homo	Pathogenic (Ib), PVS1, PM2, PM3			
72299	m	D	D	F5: NM_000130.5,c.5446C>T,p.(Pro1816Ser), het, GnomAD Gen: 0.0008						
72370	m	S	Trisomy 21	DIAPH1: NM_001079812.3,c.3200T>G,p.(Phe1067Cys), CADD: 26						Trisomy 21
72638	m	D	D	RNF213: NM_001256071.3,c.12185G>A,p.(Arg4062Gln), CADD: 31	See Table S4					
73022	f	D	D	nothing relevant						
73297	f	D	D	RNF213: NM_001256071.3,c.12374A>G,p.(Asp4125Gly), CADD 31; SPTA1: NM_003126.4,c.2600C>A,p.(Ala867Glu)	See Table S4					
73577	f	D	Mid-aortic syndrome, fructose malabsorption	RNF213: NM_001256071.3,c.5114C>A,p.(Thr1705Lys); CADD 32; RNF213: NM_001256071.3,c.3160T>A,p.(Leu1054Met); CADD 8.578; LRP1: NM_002332.3,c.2308C>T,p.(Arg770Trp); LRP1: NM_002332.3:c.3644G>A, p.(Gly1215Glu), CADD 25.9/24.7, compound het	See Table S4. Mid-aortic syndrom already associated with NF1 and RNF213 variants (PMID 29483232)					
73732	f	S	ID/DD, dysmorphic features	no variants						A. 598 Kb heterozygote deletion in chr16:29580020-30178406. Band 16p11.2. B: 2.1 Mb copy number gain (4 copies) in chr1: 145895746-148020154
73955	f	D	D	LRP1: NM_002332.3,c.8932C>T,p.(Arg2978Trp), CADD 29.2						
74316	f	S	Spherocytosis	ANK1: NM_000037.4,c.4140del,p.(Leu1382Trpfs*24); LRP1: NM_002332.3,c.7636G>A,p.(Gly2546Ser), CADD 27.3; RNF213: NM_001256071.3,c.12055C>T,p.(Arg4019Cys); CADD: 24	See Table S4	ANK1: NM_000037.4,c.4140del,p.(Leu1382Trpfs*24)	Pathogenic (Ic); PVS1, PM2, PP4			
74480	f	D	FVLeiden het, Dysmorphic features, no ID before MMA	RNF213: NM_001256071.3,c.12185G>A,p.(Arg4062Gln), CADD 31; RNF213: NM_001256071.3,c.6979A>G,p.(Asn2327Asp), rs138044665, CADD 0.049	See Table S4					
74567	m	S	NF1	<u>NF1: NM_000267.3,c.1466A>G,p.(Tyr489Cys), CADD 25.5, Clinvar: P, Intervar: VUS</u>	See Table S5	NF1: NM_000267.3,c.1466A>G,p.(Tyr489Cys)	Pathogenic (II), PS2, PS4, PP4			
74911	f	D	D	nothing relevant						
74962	m	S	NF1	NF1: NM_000267.3,c.1768del,p.(Met590Cysfs*15); MYH9: NM_002473.6,c.4396C>T,p.(Arg1466Trp), CADD: 34	See Table S5	NF1: NM_000267.3,c.1768del,p.(Met590Cysfs*15)	Pathogenic (Ic), PVS1, PM2, PP4			
75129	f	D	D	no variants						
75691	m	S	Spherocytosis	SPTB: NM_000347.5,c.2749_2750del,p.(Ser917Trpfs*86)		SPTB: NM_000347.5,c.2749_2750del,p.(Ser917Trpfs*86)	Pathogenic (Ic), PVS1, PM2, PP4			
75797	f	D	D	no variants						
76421	f	D	D	CCER2: NM_001243212.2,c.757_759del,p.(Lys253del), CADD NA, not in GnomAD						
76654	f	D	Unilateral hypoplastic bulbus oculi; no DD	ANO1: NM_018043.7,c.1276C>A,p.(His426Asn), CADD 25.1; IL12RB2: NM_001258215.1,c.973_975dup,p.(Leu325dup)						835 Kb del in chr1:10544987-11380118, genes: PEX14, CASZ1, C1orf127, TARDBP, MASP2, SRM, EXOSC10, MTOR, MTOR-AS1, ANGPTL7, UBIAD1; region: 1p36.22
76728	m	S	Hemophilia A, dysmorphic features	SPTB: NM_000347.5,c.5401C>T,p.(Arg1801Cys), CADD 34						209 Kb del in chrX:154120620-154329983, genes: F8, FUNDC2, MTCP1NB, MTCP1, BRCC3
76912	f	D	D	RNF213: NM_001256071.3,c.8212C>G,p.(Leu2738Val), rs141083276, CADD 14.42	See Table S4					
77643	f	D	D	SPTB: NM_000347.5,c.5855T>C,p.(Ile1952Thr), CADD 28.3						
78165	m	D	FVLeiden het, Dysmorphic features, no ID before MMA	LRP1: NM_002332.3,c.2308C>T,p.(Arg770Trp), CADD 25.9; NF1: NM_000267.3,c.7532C>T,p.(Ala2511Val), CADD: 23.8, benign/likely benign in Clinvar, LOVD: likely benign						
78168	m	D	D	RNF213: NM_001256071.3,c.12374A>G,p.(Asp4125Gly), CADD 31; NOTCH2: NM_024408.4,c.6169dup,p.(Asp2057Glyfs*8); NF1: NM_000267.3,c.7457C>T,p.(Thr2486Ile), rs149055633, ClinVar: VOUS, LOVD: benign	See Table S4					
78595	m	D	Morning glory anomaly right eye	PDIA4: NM_004911.5,c.296C>T,p.(Pro99Leu), het, GnomAD Ex 0.0001, CADD 33						
78715	f	S	Trisomy 21	nothing relevant						Trisomy 21
79502	f	S	NF1, macrocephaly, optic glioma, cerebellar glioma	RNF213: NM_001256071.3,c.11884A>G,p.(Asn3962Asp), CADD 13.7	See Table S4 and Table S5	Deletion of Ex1 of NF1 (detected by additional MLPA; see Table S5)	Pathogenic (Ia), PVS1, PS2, PP4	RASopathies	KAT6B: NM_001256468.2,c.2703_2714del,p.(Glu903_Glu906del), CADD: NA, AF 0.29	
79663	m	D	D	<u>ABCC6: NM_001171.6,c.2278C>T,p.(Arg760Trp), Clinvar: LP, Intervar: LP</u>, CADD 34; DNAH11: NM_001277115.2,c.6019G>A,p.(Glu2007Lys), CADD: 34						
80020	f	D	D	PKLR: NM_000298.6,c.1456C>T,p.(Arg486Trp), CADD 34						
80692	f	D	D	RNF213: NM_001256071.3,c.11998T>C,p.(Cys4000Arg), CADD 26.9						
80819	f	S	NF1	NF1: NM_000267.3,c.2970_2972del,p.(Met992del)	See Table S5	NF1: NM_000267.3,c.2970_2972del,p.(Met992del)	Likely pathogenic (II), PS4, PM2, PM4, PP4			
82059	f	S	NF1, bilateral optic gliomas, horseshoe kidneys	<u>NF1: NM_000267.3,c.7486C>T,p.(Arg2496*), Clinvar:P, Intervar:P</u>; RNF213: NM_001256071.3,c.5114C>A,p.(Thr1705Lys), CADD 32	See NF1 and Table S4	NF1: NM_000267.3,c.7486C>T,p.(Arg2496*)	Pathogenic (Ia), PVS1, PP4, PS4			
83200	m	S	NF1	nothing relevant		Deletion of Ex 28 and 29 of NF1 (detected by additional MLPA; see Table S5)	Pathogenic (Id), PVS1, PP1, PP4	RASopathies	ACTB: NM_001101.5,c.-6-4G>T, rs13447394, Clinvar: benign (2 stars)	
83661	f	D	D	RPTN: NM_001122965.1,c.1375C>T,p.(Gln459*), CADD 27.7						
83831	f	S	FMD, autoimmune, Livido racemosa	ANO1: NM_018043.7,c.2410G>A,p.(Val804Met), CADD 32						
83955	m	D	D	ZNF783: NM_001195220.2,c.378G>T,p.(Trp126Cys), CADD 26.8						
84522	m	S	NF1	NF1: NM_000267.3,c.1541_1542del,p.(Gln514Argfs*43); ANK1: NM_000037.4,c.4448T>G,p.(Leu1483Arg)	See Table S5	NF1: NM_000267.3,c.1541_1542del,p.(Gln514Argfs*43)	Pathogenic (Ia), PVS1, PS2, PM2, PP4, PP5			
84982	m	S	Alagille	<u>JAG1: NM_000214.3,c.1156G>A,p.(Gly386Arg), CADD 34, Clinvar: LP, Intervar: LP; LRP1: NM_002332.3,c.176A>C,p.(Glu59Ala), CADD: 28.9, Clinvar: unknown, Intervar: LP</u>; LRP1: NM_002332.3,c.3644G>A,p.(Gly1215Glu), CADD: 24.7; SLC4A1: NM_000342.4,c.2401A>C,p.(Ser801Arg), CADD 27		JAG1: NM_000214.3,c.1156G>A,p.(Gly386Arg)	Pathogenic (II), PS3, PS4, PM2, PP4			
85069	f	S	ACTA2	<u>ACTA2: NM_001141945.3,c.536G>A,p.(Arg179His), CADD 25.1, Clinvar: P, Intervar: VUS</u>; RNF213: NM_001256071.3,c.14194A>G,p.(Lys4732Glu), CADD 4	See Table S4	ACTA2: NM_001141945.3,c.536G>A,p.(Arg179His)	Pathogenic (II), PS3, PS4, PM1, PM2			
86942	f	D	D	nothing relevant						
87022	f	D	D	NOTCH2: NM_001200001.2,c.3625T>G,p.(Phe1209Val), benign/likely benign in Clinvar (2 stars, rs147223770)						
87654	m	S	Short stature, DD (possibly secundary to MMA)	RNF213: NM_001256071.3,c.9952A>G,p.(Ile3318Val), rs147785564, CADD 0.06	See Table S4					
87663	m	D	D	SBF2: NM_030962.4,c.5014_5016del,p.(Lys1672del), CADD: NA; <u>MYH11: NM_002474.3,c.1502G>A,p.(Arg501His), CADD 34, Clinvar: conflicting interpretations, Intervar: LP</u>; MYH9: NM_002473.6,c.691A>G,p.(Asn231Asp), CADD: 28.3						
87885	f	D	D	SGCE: NM_001301139.2,c.433G>A,p.(Ala145Thr), rs746585235, Benign in ClinVar; MYH9: NM_002473.6,c.4396C>T,p.(Arg1466Trp), CADD: 34						
88103	f	S	NF1	<u>NF1: NM_000267.3,c.574C>T,p.(Arg192*), CADD 36, Clinvar:P, Intervar: P</u>; ELN: NM_001278913.2,c.1083_1084del,p.(Tyr361*); SBF2: NM_030962.4,c.1967G>C,p.(Cys656Ser), CADD 27.7	See Table S5	NF1: NM_000267.3,c.574C>T,p.(Arg192*)	Pathogenic (Ia), PVS1, PS4, PP4			
88884	f	S	NF1	NF1: NM_000267.3,c.4971_4977del,p.(Tyr1657*), PLOD1: NM_000302.4,c.1927G>A,p.(Val643Ile), CADD: 34	See Table S5	NF1: NM_000267.3,c.4971_4977del,p.(Tyr1657*)	Pathogenic (Ia), PVS1, PS2, PM2, PP4			
88905	m	S	MOPD2	<u>PCNT: NM_001315529.2,c.7450G>T,p.(Glu2484*), homo, CADD: 43, Clinvar: P, Intervar: P</u>; PALD1: NM_014431.3,c.1549_1553dup,p.(Gln519Argfs*16), CADD: NA; MYH11: NM_002474.3,c.5676G>C,p.(Glu1892Asp), CADD 27.3; MTFMT: NM_139242.4,c.172T>A,p.(Phe58Ile), CADD: 25.3		PCNT: NM_001315529.2,c.7450G>T,p.(Glu2484*)	Pathogenic (Ib), PVS1, PM2, PM3, PP4			
90045	m	D	D	PDIA4: NM_004911.5,c.1576C>T,p.(Pro526Ser), CADD 25						
90048	f	D	D	SPTA1: NM_003126.4,c.1489G>T,p.(Ala497Ser), CADD 25.7						
90101	m	D	D	RNF213: NM_001256071.3,c.9668C>T,p.(Ser3223Leu); CADD 34						
90549	m	D	Stenosis of the aortic isthmus	nothing relevant						
91071	m	D	D	<u>RNF213: NM_001256071.3,c.14429G>A,p.(Arg4810Lys), Clinvar: P, Intervar: VUS</u>; RNF213: NM_001256071.3,c.14194A>G,p.(Lys4732Glu), CADD: 4;CD46: NM_002389.4,c.811_816del,p.(Asp271_Ser272del); SLC4A1: NM_000342.4,c.2116C>A,p.(Leu706Met), CADD: 25.9; NOTCH2: NM_001200001.2,c.17_18del,p.(Pro6Argfs*27), CADD: NA						
92357	f	D	D	nothing relevant						
93045	f	S	ACTA2	<u>ACTA2: NM_001141945.3,c.536G>A,p.(Arg179His), CADD 25.1, Clinvar: P, Intervar: VUS</u>		ACTA2: NM_001141945.3,c.536G>A,p.(Arg179His)	Pathogenic (II), PS3, PS4, PM1, PM2			
93051	f	D	D	no variants						
94029	m	D	D	<u>RNF213: NM_001256071.3,c.12040C>T,p.(His4014Tyr), CADD 29.7, Clinvar: unknown, Intervar: LP</u>; RNF213: NM_001256071.3,c.6979A>G,p.(Asn2327Asp), CADD: 0.049; RNF213: NM_001256071.3,c.14194A>G,p.(Lys4732Glu), CADD: 4; DIAPH1: NM_001079812.3,c.1742G>T,p.(Gly581Val); CADD: 24.5	See Table S4					
94084	f	S	NF1, optic gliomas, multiple neurofibromas	NF1: NM_000267.3,c.6908del,p.(Gln2303Argfs*22), CADD: NA; MYH11: NM_002474.3,c.5226G>C,p.(Glu1742Asp), CADD: 29,1		NF1: NM_000267.3,c.6908del,p.(Gln2303Argfs*22)	Pathogenic (II), PS2, PS4, PS3, PM2, PP4, PP3			
94488	m	S	NF1	NF1:NM_000267:c.1642-1359A>T†; c.8315-5214A>G†; NOTCH2: NM_001200001.2,c.17_18del,p.(Pro6Argfs*27), CADD NA; <u>ABCC6: NM_001171.6,c.3823C>T,p.(Arg1275*), CADD: 38, Clinvar: LP, Intervar: P</u>; HDAC9: NM_001204147.3,c.692G>A,p.(Arg231His), CADD 26.8		NF1:NM_000267.3:c.1642-1359A>T†; c.8315-5214A>G†	Likely benign (I), BS2, BP4 / VUS, BS2	RASopathies	SOS1: NM_005633.4,c.2371C>A,p.(Leu791Ile), rs142004123, Clinvar: benign (3 star)	
94566	f	D	D	NOTCH2: NM_001200001.2,c.17_18del,p.(Pro6Argfs*27), CADD NA; SBF2: NM_030962.4,c.2054A>C,p.(Tyr685Ser), CADD 29 (GWAS gene); DIAPH1: NM_001079812.3,c.1742G>T,p.(Gly581Val); CADD: 24.5						
95246	m	D	D	RNF213: NM_001256071.3,c.14194A>G,p.(Lys4732Glu), CADD: 4; DNAH5: NM_001369.3,c.1126G>T,p.(Ala376Ser), CADD: 24.7	See Table S4					
95538	f	S	Trisomy 21, Evans syndrome	nothing relevant						Trisomy 21
95540	f	S	NF1, macrocephaly	NF1:NM_000267: c.6579+18A>G†		NF1:NM_000267.3: c.6579+18A>G†	Pathogenic (II), PS2, PS4, PS3, PM2, PP4	RASopathies	No variants	
96282	f	D	D	nothing relevant						
97355	m	S	COL4A2-related brain small vessel disease type 2, poroencephalic cyst in MRI	no variants		COL4A2: NM_001846.4, c.3634G>A, p.(Gly1212Ser), de novo (gene not present in Moyamoya filter)	Likely pathogenic (II), PM2, PP3, PP4, PS2			
97764	f	D	D	nothing relevant						
98340	m	D	D	RNF213: NM_001256071.3,c.12345A>C,p.(Lys4115Asn), CADD 25.1; SPTB: NM_000347.5,c.5926G>A,p.(Ala1976Thr), CADD34						
98932	f	S	NF1	NF1: NM_000267.3,c.3739_3742del,p.(Phe1247Ilefs*18); CLN6: NM_017882.3,c.700T>C,p.(Phe234Leu), CADD 24.4	See Table S5	NF1: NM_000267.3,c.3739_3742del,p.(Phe1247Ilefs*18)	Pathogenic (Ia), PVS1, PS2, PM2, PP4, PP5			
99265	m	D	Dysmorphic features, no DD	NOTCH2: NM_001200001.2,c.17_18del,p.(Pro6Argfs*27)						
100129	m	D	D	RNF213: NM_001256071.3,c.11998T>C,p.(Cys4000Arg), CADD 26.9; PCNT: NM_001315529.2,c.4348G>A,p.(Glu1450Lys), CADD 25.7; ADAMTS13: NM_139025.5,c.559G>C,p.(Asp187His), CADD 25.6; RNF213: NM_001256071.3,c.6551A>G,p.(Gln2184Arg)						
100132	f	D	D	MTFMT: NM_139242.4,c.172T>A,p.(Phe58Ile), CADD 25.3						
75558	m	S	Ocular apraxia, cerebellar anomalies, ataxia, DD: lower-normal range, obesity	FANCC: NM_000136.3,c.67del,p.(Asp23Ilefs*23); ADAMTS13: NM_139025.5,c.905G>A,p.(Gly302Asp), CADD25.8						
76572	f	D	vertebral artery stenosis, possible FMD	nothing relevant						
78053	m	D	D	RNF213: NM_001256071.3,c.13913C>T,p.(Thr4638Ile), CADD 24.6						
78603	m	S	T1DM, Hypothyroidism, GH-Deficiency, short stature, IgA-deficiency, microcephaly	SLC7A7: NM_001126105.3,c.1382_1384del,p.(Ile461del); RNF213: NM_001256071.3,c.6350G>A,p.(Arg2117His), CADD 2.7; MYH9: NM_002473.6,c.4298G>A,p.(Arg1433His), CADD: 32	See Table S4					
81910	m	D	D	ELN: NM_001278913.2,c.1221_1274del,p.(Leu416_Gly433del)						
84980	f	D	D	RNF213: NM_001256071.3,c.14546T>A,p.(Ile4849Asn), CADD 24.9	See Table S4					
99799	f	D	D	RNF213: NM_001256071.3,c.1214_1216del,p.(Gly405del), CADD: 12.75; RNF213: NM_001256071.3,c.12341C>A,p.(Thr4114Lys), CADD 34, <u>MTHFR: NM_001330358.2,c.1127G>A,p.(Arg376His), CADD27.9, Clinvar: P, Intervar: LP</u>; NOTCH2: NM_001200001.2,c.17_18del,p.(Pro6Argfs*27), CADD: NA; F5: NM_000130.5,c.112G>T,p.(Ala38Ser), CADD: 26.8, PCNT: NM_001315529.2,c.1800G>A,p.(=), CADD: NA; PCNT: NM_001315529.2,c.4351_4353del,p.(Glu1451del), CADD: NA ; DNAH11: NM_001277115.2,c.8572G>A,p.(Gly2858Ser), CADD: 34	See Table S4					
